# Therapeutic siRNA: state of the art

**DOI:** 10.1038/s41392-020-0207-x

**Published:** 2020-06-19

**Authors:** Bo Hu, Liping Zhong, Yuhua Weng, Ling Peng, Yuanyu Huang, Yongxiang Zhao, Xing-Jie Liang

**Affiliations:** 1 School of Life Science, Advanced Research Institute of Multidisciplinary Science, Institute of Engineering Medicine, Key Laboratory of Molecular Medicine and Biotherapy, Beijing Institute of Technology, 100081 Beijing, People’s Republic of China; 2 National Center for International Biotargeting Theranostics, Guangxi Key Laboratory of Biotargeting Theranostics, Collaborative Innovation Center for Targeting Tumor Theranostics, Guangxi Medical University, 530021 Guangxi, People’s Republic of China; 3 Aix-Marseille University, CNRS, Centre Interdisciplinaire de Nanoscience de Marseille (CINaM), Equipe Labellisée Ligue Contre le Cancer, 13288 Marseille, France; 4 Chinese Academy of Sciences (CAS), Key Laboratory for Biomedical Effects of Nanomaterials and Nanosafety, CAS Center for Excellence in Nanoscience, National Center for Nanoscience and Technology of China, 100190 Beijing, People’s Republic of China

**Keywords:** Nucleic-acid therapeutics, Oligo delivery, Drug delivery, Drug delivery, Gene therapy

## Abstract

RNA interference (RNAi) is an ancient biological mechanism used to defend against external invasion. It theoretically can silence any disease-related genes in a sequence-specific manner, making small interfering RNA (siRNA) a promising therapeutic modality. After a two-decade journey from its discovery, two approvals of siRNA therapeutics, ONPATTRO^®^ (patisiran) and GIVLAARI™ (givosiran), have been achieved by Alnylam Pharmaceuticals. Reviewing the long-term pharmaceutical history of human beings, siRNA therapy currently has set up an extraordinary milestone, as it has already changed and will continue to change the treatment and management of human diseases. It can be administered quarterly, even twice-yearly, to achieve therapeutic effects, which is not the case for small molecules and antibodies. The drug development process was extremely hard, aiming to surmount complex obstacles, such as how to efficiently and safely deliver siRNAs to desired tissues and cells and how to enhance the performance of siRNAs with respect to their activity, stability, specificity and potential off-target effects. In this review, the evolution of siRNA chemical modifications and their biomedical performance are comprehensively reviewed. All clinically explored and commercialized siRNA delivery platforms, including the GalNAc (*N*-acetylgalactosamine)–siRNA conjugate, and their fundamental design principles are thoroughly discussed. The latest progress in siRNA therapeutic development is also summarized. This review provides a comprehensive view and roadmap for general readers working in the field.

## Introduction

Gene therapy is a promising therapeutic platform because it targets disease-causing genes in a sequence-specific manner, which enables more precise and personalized treatment of diverse life-threatening diseases.^1^ By introducing a certain nucleic acid modality to the desired tissue of the patient, gene expression can be downregulated, augmented or corrected. Small interfering RNA (siRNA), microRNA (miRNA) and inhibitory antisense oligonucleotides (ASOs) are representative molecules used to trigger gene inhibition, whereas plasmid DNA, messenger RNA (mRNA), small activating RNA (saRNA), splicing-modulatory ASOs and CRISPR (clustered regularly interspaced short palindromic repeats)/Cas (CRISPR-associated protein) systems are usually employed to increase or correct target gene expression.^2–4^ Currently, many therapeutic programs have been explored to treat certain diseases.

**Fig. 1:**
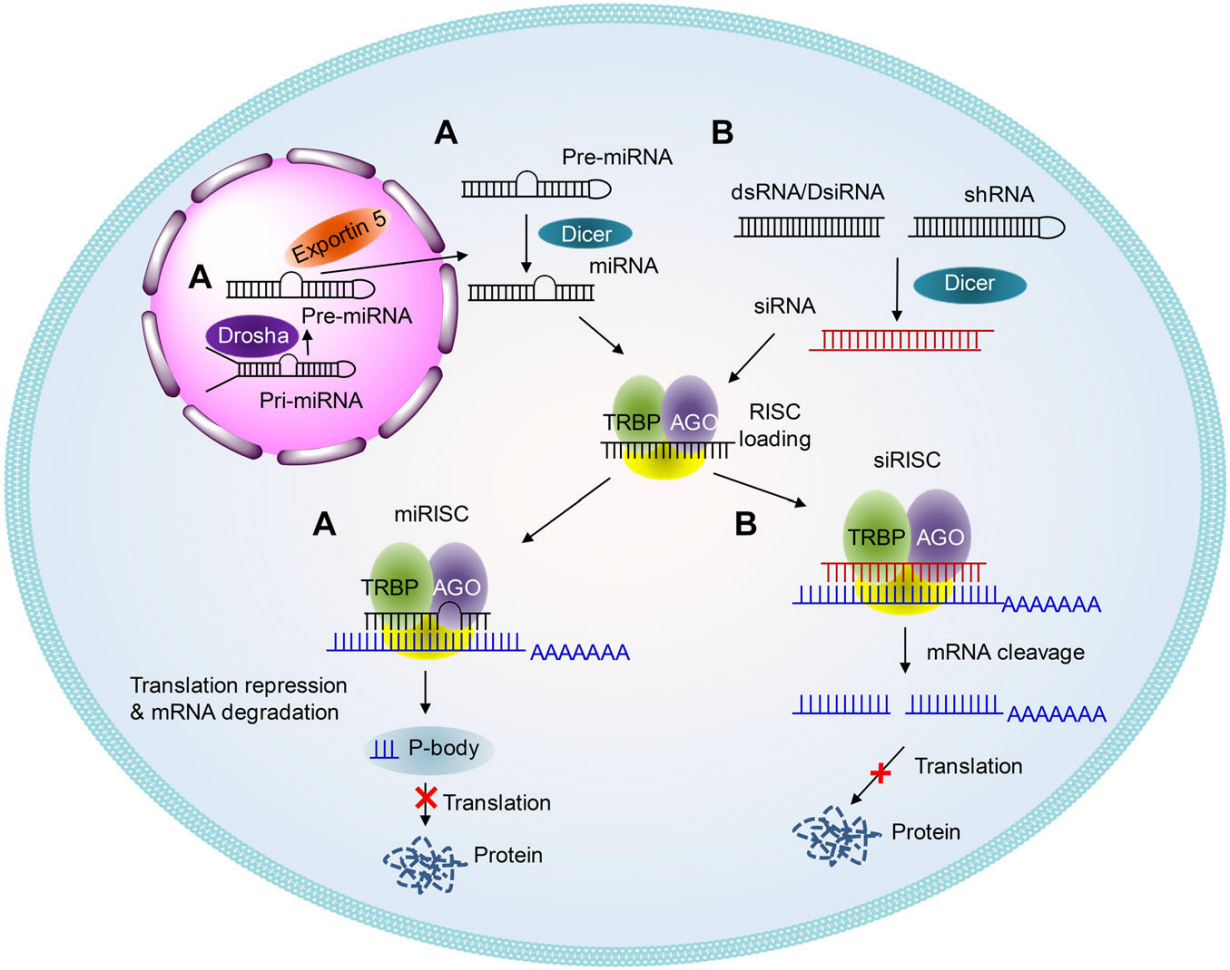
Schematic illustrations of the working mechanisms of miRNA (**a**) and siRNA (**b**)

RNA interference (RNAi) is a natural defense mechanism for the invasion of exogenous genes.^5,6^ RNAi modalities, e.g., siRNA and miRNA, can knockdown the expression of target genes in a sequence-specific way (Fig. 1) by mediating targeted mRNA degradation (for siRNA and miRNA) or mRNA translation repression (for miRNA). As a result of the slight differences between siRNA and miRNA, siRNA can typically trigger more efficient and specific gene silencing than miRNA, whereas one miRNA may compromise the expression of several different target genes simultaneously. Hence, siRNA and miRNA have different roles in pharmaceutical practice.

Since the establishment of the RNAi concept in 1998, siRNA therapeutics have experienced many ups and downs. In 2001, Tuschl et al.^7^ successfully silenced the expression of a specific gene by introducing chemically synthesized siRNA into mammalian cells, leading to the emergence of a developmental upsurge. Although siRNA therapy once suffered due to the obstacles of its stability, specificity and delivery, advances in chemical modification and delivery brought the field to a robust and rapidly developing area of research again in recent years. After a 20-year journey, the United States Food and Drug Administration (FDA) and the European Commission (EC) approved ONPATTRO^®^ (patisiran, ALN-TTR02) as the first commercial RNAi-based therapeutic for the treatment of hereditary amyloidogenic transthyretin (hATTR) amyloidosis with polyneuropathy in adults in 2018.^2,8^ Recently, the FDA-approved GIVLAARI™ (givosiran, ALN-AS1) for the treatment of adults with acute hepatic porphyria (AHP).^9–12^

siRNA has innate advantages over small molecular therapeutics and monoclonal antibody drugs because siRNA executes its function by complete Watson–Crick base pairing with mRNA, whereas small molecule and monoclonal antibody drugs need to recognize the complicated spatial conformation of certain proteins. As a result, there are many diseases that are not treatable by small molecule and monoclonal antibody drugs since a target molecule with high activity, affinity and specificity cannot be identified. In contrast, theoretically, any gene of interest can be targeted by siRNA since only the right nucleotide sequence along the targeting mRNA needs to be selected. This advantage confers the siRNA modality with a shorter research and development span and a wider therapeutic area than small molecule or antibody drugs, especially for those genes that are unfeasible for development with such strategies.

Although siRNA holds promising prospects in drug development, several intracellular and extracellular barriers limit its extensive clinical application. Naked and unmodified siRNA possesses some disadvantages, such as (1) unsatisfactory stability and poor pharmacokinetic behavior and (2) the possible induction of off-target effects. The phosphodiester bond of siRNA is vulnerable to RNases and phosphatases. Once it is systematically administered into circulation, endonucleases or exonucleases throughout the body will quickly degrade siRNA into fragments, thus preventing the accumulation of intact therapeutic siRNA in the intended tissue. In theory, siRNA only functions when its antisense strand is completely base-paired to the target mRNA. However, a few mismatches are tolerated by the RNA-induced silencing complex (RISC), which may lead to undesired silencing of those genes with a few nucleotide mismatches. In addition, the RISC-loaded sense strand of siRNA may also knockdown the expression of other irrelevant genes. Moreover, unformulated and unmodified siRNA may lead to the activation of Toll-like receptor 3 (TLR3) and adversely affect the blood and lymphatic systems.^13^ These discoveries have raised many concerns about the undesirable effects and pharmaceutical issues of siRNAs.

To maximize the treatment potency and reduce or avoid the side effects of siRNA, researchers have made great efforts to investigate various chemical modification geometries and to develop many different delivery systems. As a result, a series of modification patterns were proposed and evaluated preclinically and clinically with respect to their effects on activity, stability, specificity and biosafety. Delivery materials derived from lipids, lipid-like materials (lipidoids), polymers, peptides, exosomes, inorganic nanoparticles, etc., have been designed and investigated.^2,8,14–22^ As a result, several modification patterns and delivery platforms have been employed in clinical studies. Here, the detailed evolution and advances in the modification and delivery technologies of siRNA are comprehensively summarized and discussed. This review provides an overview and a handbook for reviewing siRNA therapeutic development.

## siRNA modification

During the early stages of developing siRNA therapeutics, many agents were designed based on completely unmodified or slightly modified siRNA to arrive at appropriate tissue and then silence the target gene. These molecules can mediate gene silencing in vivo, especially in tissues that receive local drug administration, e.g., eyes. However, limited efficacy and potential off-target effects may be observed with these modalities. For example, Kleinman and colleagues^23^ observed that bevasiranib and AGN211745 triggered significant activation of toll-like receptor 3 (TLR3) and its adapter molecule TRIF, inducing the secretion of interleukin-12 and interferon-γ. Bevasiranib and AGN211745 are siRNA therapeutics developed for the treatment of age-related macular degeneration.^24^ The VEGFA-targeting bevasiranib^25^ and VEGFR1-targeting AGN211745^26^ are unmodified and slightly modified siRNAs, respectively. Moreover, Kleinman and colleagues further demonstrated that siRNA classes with sequences of 21 nucleotides or longer, regardless of which genes they target, can suppress CNV in mice compared to bevasiranib and AGN211745. These findings eventually led to the termination of the clinical investigation of bevasiranib in 2009.

**Fig. 2:**
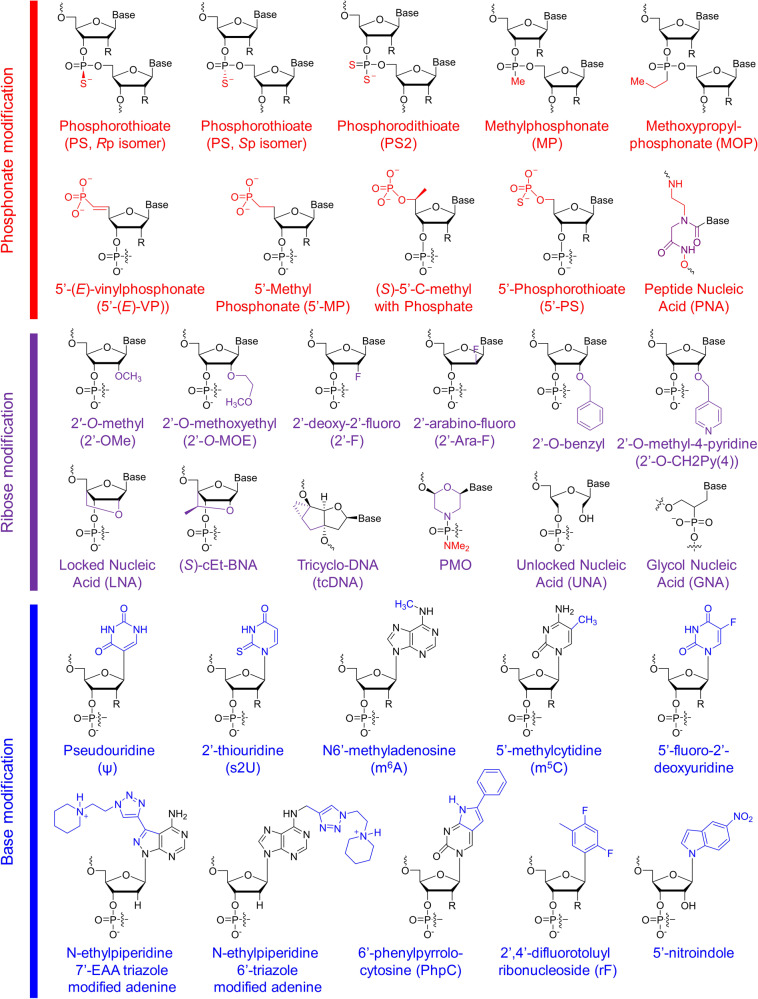
Structures of chemical modifications and analogs used for siRNA and ASO decoration. According to the modification site in the nucleotide acid, these structures can be divided into three classes: phosphonate modification, ribose modification and base modification, which are marked in red, purple and blue, respectively. R = H or OH, for RNA or DNA, respectively. (*S*)-cEt-BNA (*S*)-constrained ethyl bicyclic nucleic acid, PMO phosphorodiamidate morpholino oligomer

Chemically modified siRNAs, such as siRNAs with substitution of the 2′-OH with a 2′-O-methyl (2′-OMe)^27^ or 2′-methoxyethyl (2′-MOE)^28^ group or the substitution of certain nucleotides with locked nucleic acid (LNA),^29^ unlocked nucleic acid (UNA)^30^ or glycol nucleic acid (GNA)^31^ (Fig. 2), can efficiently suppress immunostimulatory siRNA-driven innate immune activation, enhance activity and specificity, and reduce off-target-induced toxicity. To enhance the potency and reduce the potential toxicity of siRNA, numerous chemical modification geometries have been established and tested.^32^

**Fig. 3:**
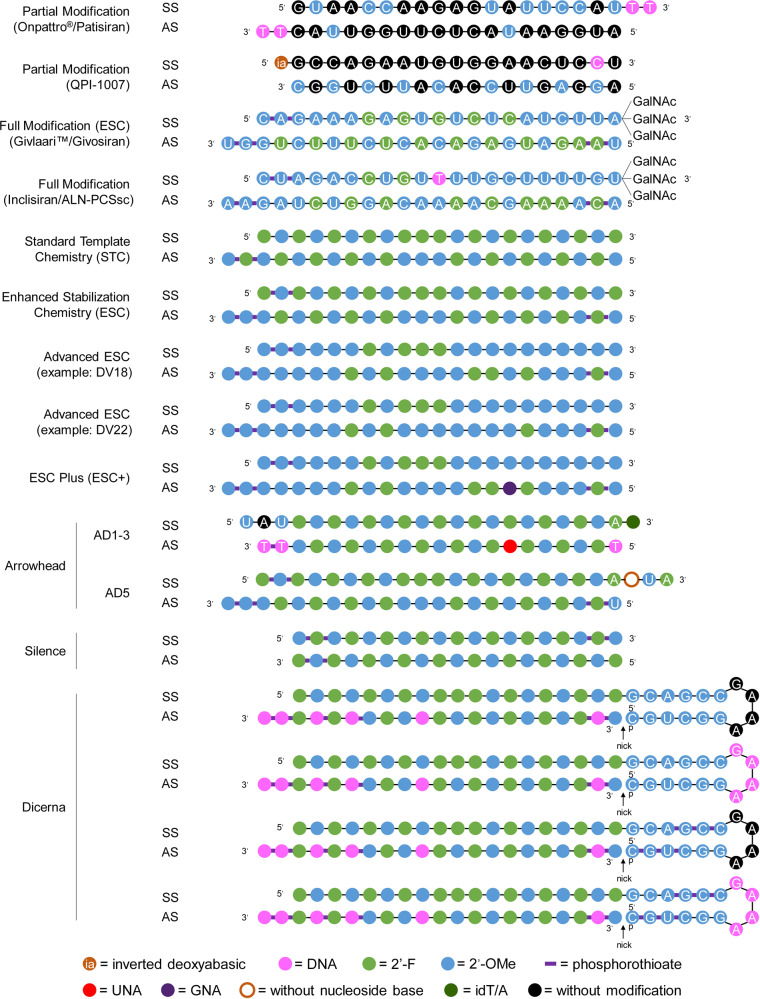
Representative designs for the chemical modification of siRNA. The sequences and modification details for ONPATTRO^®^, QPI-1007, GIVLAARI™ and inclisiran are included. The representative siRNA modification patterns developed by Alnylam (STC, ESC, advanced ESC and ESC+) and arrowhead (AD1-3 and AD5) are shown. Dicerna developed four GalNAc moieties that can be positioned at the unpaired G–A–A–A nucleotides of the DsiRNA structure. 2′-OMe 2′-methoxy, 2′-F 2′-fluoro, GNA glycol nucleic acid, UNA unlocked nucleic acid, SS sense strand, AS antisense strand

According to the natural structure of nucleotides, chemical modifications can be placed at the phosphate backbone, the ribose moiety or the base. Typically, these modifications are simultaneously introduced in siRNA. For example, the combination of 2′-OMe and phosphorothioate (PS) modifications facilitates the systemic administration of cholesterol-conjugated siRNA and achieves efficient gene silencing in vivo.^33^ In addition, the combination of 2′-OMe and 2′-deoxy-2′-fluoro (2′-F) has been used for ONPATTRO^®^^34,35^ (Figs. 2, 3). For QPI-1007, a naked and unformulated siRNA therapeutic,^2^ 2′-OMe was alternatively incorporated into the antisense strand, and an inverted deoxyabasic moiety and an l-DNA cytidine nucleotide were used in the sense strand^36^ (Figs. 2,3). Moreover, modifications with phosphorothioate (PS), 2′-OMe, 2′-F and 2′-deoxy were employed for inclisiran (ALN-PCSsc), an siRNA therapeutic for the treatment of hypercholesterolemia^37,38^ (Figs. 2, 3). Overall, the precise modification of siRNA can increase its efficacy, specificity, and stability and reduce its toxicity and immunogenicity. Detailed information will be discussed in the following sections.

### Phosphonate modification

Phosphorothioate (PS) linkage has previously been used in antisense oligonucleotide (ASO) modification.^39^ This modification was achieved by leveraging a sulfur atom to replace one nonbridging oxygen of a phosphodiester (Fig. 2). PS linkage endows modified oligonucleotides with resistance to nucleases,^40^ and these molecules more readily combine with plasma proteins,^41^ especially albumin^42^ than their unmodified counterparts, which may result in a longer circulation time. Although some data have shown that PS modification reduces the binding affinity between the oligonucleotide and its target sequence^32^ to some extent and aggravates chemistry-related toxicities with a high PS content,^43,44^ PS modification is still very important and necessary for both siRNA and ASO because, compared with unmodified oligonucleotides, PS-modified oligonucleotides are more hydrophobic and stable molecules with higher affinity to certain proteins.^44^ Appropriate protein binding may prolong the half-life of oligonucleotides in circulation, and the half-life of the elimination phases may be as long as a few days.^41^ Protein binding is typically beneficial for the cell entry of oligonucleotides; however, too much protein binding showed a positive correlation with in vivo toxicity.^45,46^ Hence, the position and number of PS linkages are pivotal for their application. For siRNA modification, Alnylam introduced two PS linkages at the first two nucleotides at the 5′-end of the sense strand and two PS linkages at the first two nucleotides at both the 5′-end and the 3′-end of the antisense strand of siRNA (Fig. 3). Moreover, compared to the parent siRNA without modifications, the PS linkage-containing siRNA has a barely changed in vivo biodistribution profile, as it predominantly accumulates in clearance organs, e.g., liver, kidneys and intestines, followed by glandular tissues, bone marrow, adipocytes and lymph nodes.^47–50^

Notably, there are two configurations for PS linkages, namely, *R*p and *S*p isomers (Fig. 2). Verdine and colleagues (team from Wave Life Sciences)^51^ demonstrated that the stereochemistry of PS significantly influences the pharmacologic performance of ASOs. PS linkages with the *S*p configuration are more stable than those with the *R*p configuration. More importantly, they unveiled that stereopure ASOs with a triplet stereochemical code, 3′-*S*p*S*p*R*p, make the target RNA more vulnerable to RNase H1 in vitro and achieve a more durable inhibition profile in vivo than stereorandom ASOs. However, Østergaard et al. (team from Ionis Pharmaceuticals)^52^ disclosed that controlling PS chirality markedly affects the interactions between the ASOs and RNase H1; however, this modulation failed to enhance the overall therapeutic effects. The sequence and design of ASOs are the primary drivers that determine the performances of gapmer ASOs. In addition, recently disclosed patents filed by Alnylam reported that the chirality of PS linkages may markedly influence siRNA performance.^53^ The terminal chiral PS linkages at the 3′- and 5′-ends of the antisense strand of siRNA prefer *S*p and *R*p configurations, respectively. The chiral PS number can be 1, 2 or 3 at the 3′-end and 1 or 2 at the 5′-end of the siRNA antisense strand. In addition, the chiral PS linkages at the 5′-end of the siRNA sense strand can adopt either an *S*p or an *R*p configuration and are positioned at the first internucleotide linkage at the 5′-end of the sense strand. Overall, the chirally modified siRNA can comprise four, five or more terminal, chirally modified PS linkages.

In addition, other residues have been identified and successfully used to replace the phosphodiester group in oligonucleotides and change the properties of intact strands, including phosphorodithioate (PS2),^54^ methylphosphonate (MP), methoxypropylphosphonate (MOP) and peptide nucleic acid (PNA) (Fig. 2). PS2 modification can increase the affinity between RISC and siRNA. The site-specific incorporation of MP and MOP modifications has been used to reduce ASO protein binding since the PS backbone is negatively charged, whereas alkylphosphonate linkages are charge neutral. As a result, the MOP linkage incorporated at position 2 or 3 from the 5′-end of the DNA gap significantly mitigated the hepatotoxicity of ASOs.^46^ In addition, both PNA^55–57^ (Fig. 2) and phosphotriesters^58^ are very meaningful modifications to make siRNA or ASO molecules more druggable, although these modifications are not as predominant as PS. PNA is also typically used to modify detection probes for capturing and detecting certain nucleic acid targets.^59^

In addition, phosphonate modification with various analogs at the 5′-end of siRNA or single-stranded siRNA (ss-siRNA) is a newly developed but important decoration strategy to enhance siRNA activity. The 5′-phosphate of exogenously introduced siRNA is required for RISC loading. A 5′-phosphate can be modified via either phosphorylation in cells mediated by Clp1 (cleavage and polyadenylation factor I subunit 1) or chemical synthesis in the laboratory.^60^ However, the natural 5′-phosphate can also be removed by dephosphorylation in cells. Together with the demand to enhance siRNA stability, researchers have identified a series of analogs that have similar conformation and steroidal electronic properties to natural phosphates but are resistant to dephosphorylases. As a result, 5′-(*E*)-vinyl phosphonate (5′-(*E*)-VP), 5′-methylphosphonate, (*S*)-5′-C-methyl with phosphate, 5′-phosphorothioate, etc., were designed and evaluated in vitro and in vivo^61^ (Fig. 2). Among these strategies, 5′-(*E*)-VP decoration, which substitutes bridge oxygen and carbon with *E*-vinyl phosphonate moieties at the 5′-end, is the most potent and metabolically stable mechanism. The replaced hydrophobic substitutions enhance the stability of intact oligonucleotides and are favorable for RISC loading. Ionis Pharmaceuticals first developed and own the intellectual property rights to the 5′-(*E*)-VP structure. This technology was first applied to ss-siRNA and achieved ideal pharmacodynamic and pharmacokinetic profiles. Modifying ss-siRNA with 5′-(*E*)-VP and other specific modification structures enables potent repression of targeted gene expression by ss-siRNA in vivo.^61,62^ Inspired by this finding, the 5′-(*E*)-VP modification was further applied to double-stranded siRNA. Data have revealed that this modification improves siRNA accumulation and residence time in tissue and enhances siRNA potency in vivo by elevating Argonaute-2 binding.^63–65^

### Ribose modification

Ribose modifications at the 2′ position have been widely used to protect siRNA from attacking ribonucleases, which require the 2′-OH group for the hydrolysis of RNA. Notably, 2′-O-methyl (2′-OMe) (Fig. 2) is a naturally occurring ribosugar and the most frequently used modification in drug development so far^66^ because this molecule can enhance stability^67^ by blocking the nucleophilic 2′-OH group. In addition, 2′-OMe also shows increased affinity for its target mRNA^68^ and reduced immunogenicity in the body. Based on 2′-OMe, a series of analogs have been identified, among which 2′-O-methoxyethyl (2′-O-MOE) (Fig. 2) is one of the most useful and popular analogs of 2′-OMe. Moreover, 2′-O-MOE shows higher binding affinity for RNA than 2′-OMe with a 0.9–1.7 °C change in Δ*T*_m_ per modified nucleotide, which further increases the capability of the modified siRNA to resist nuclease attack.^32^ Another widely used 2′-OH substitution is 2′-deoxy-2′-fluoro (2′-F)^69^ (Fig. 2). Highly electronegative fluorine makes the modified siRNA readily adopt a C3′-endo conformation, providing considerable benefits in binding affinity, with a Δ*T*_m_ of ~2.5 °C per modified nucleotide.^32^ Interestingly, 2′-arabino-fluoro (2′-Ara-F), an alternative fluoro substitution form, can also be used to modify nucleic acid therapeutics, e.g., antisense oligonucleotides^45,70^ and siRNA.^71^ Both 2′-O-benzyl and 2′-O-methyl-4-pyridine (2′-O-CH2Py(4)) were well tolerated when they were incorporated on the guide strand of siRNA in vivo^72^ (Fig. 2). siRNA with modifications of four 2′-O-benzyl or six 2′-O-CH2Py(4) showed comparable activity with the unmodified siRNA in vivo, and increased activity was even achieved when these modifications were placed at the 8 and 15 positions on the siRNA guide strand.

In addition, 2′-C, 4′-C, and even the whole sugar ring can also undergo modifications, resulting in molecules including UNAs, LNAs, GNAs, (S)-cEt-BNAs, tricyclo-DNA (tcDNA) and phosphorodiamidate morpholino oligomers (PMOs) (Fig. 2). UNA, with higher flexibility and thermal destabilization than the unmodified product due to unconnected 2′ and 3′ carbons, can block the entry of passenger strands and promote the RISC loading of the guide strand by introducing chemical asymmetry into duplex siRNAs. Similarly, glycol nucleic acid (GNA),^31^ another thermally destabilizing nucleotide, can be used to erase off-target effect-induced hepatotoxicity by including it in the seed region of the siRNA guide strand. LNA is a bicyclic structure that contains a bridge between the 2′ oxygen and the 4′ carbon. It “locks” the ribose into its preferred C3′-*endo* conformation and significantly increases the affinity of base pairing.^40^ Data have shown that the incorporation of LNAs increases the DNA melting temperature up to 8 °C per LNA.^73^ Given the successes of LNAs, many more bicyclic and even tricyclic analogs, including ethyl-bridged nucleic acids (ENAs),^74^ constrained ethyl (cEt) nucleic acids^75^ and tricyclo-DNA,^76^ have been engineered and incorporated into RNA or DNA strands in succession. Moreover, PMOs^77^ do not look like classic nucleotides, as the ribose subunit has been substituted with a morpholine subunit. PMOs are uncharged at physiological pH and are not substrates of RNase H. Therefore, they are mainly used to block RNA splicing or translation.^78^

### Base modification

Base replacement is of great benefit to nucleic acid-based drug development. For instance, the substitution of pseudouridine,^79^ 2-thiouridine,^80^ N6-methyladenosine,^80^ 5-methylcytidine^81^ (Fig. 2) or other base analogs of uridine and cytidine residues can reduce innate immune recognition while making ASOs more resistant to nucleases. However, the artificial base substitution of ASOs, similar to siRNA, is basically at the stage of research and development. Pharmaceutical corporations still hold a prudent attitude toward these molecules. Instead, these companies prefer to use naturally occurring base structures, e.g., 5mC and 6 mA, to modify certain base(s), probably because of concerns about the safety of the metabolized unnatural residues that potentially might be incorporated into the genome.

*N*-ethylpiperidine triazole-modified adenosine analogs with Hoogsteen or Watson–Crick (WC) facial localization have also been used to modify siRNA,^82–85^ which can disrupt nucleotide/TLR8 interactions and therefore reduce the immunogenicity of siRNA. *N*-ethylpiperidine 7-EAA triazole (7-EAA, 7-ethynyl-8-aza-7-deazaadenosine) can pair well with uridine and form an A-form helix structure. This molecule will be recognized as adenosine by Avian myeloblastosis virus reverse transcriptase in RNA strands.^83^

In addition, 6′-phenylpyrrolocytosine (PhpC) is a cytosine mimic showing excellent base pairing fidelity, thermal stability and high fluorescence.^86^ PhpC-containing siRNA shows gene-silencing activity comparable to that of the parent molecule, and its fluorescent properties make it useful for fluorescence-based detection or monitoring or for exploring the cellular uptake and trafficking of siRNA in cells.

The internal uridine substitution of 2,4-difluorotolylribonucleoside (rF) is well tolerated by siRNA activity.^87^ The base pairing between rF and adenosine (A) is relatively unstable compared to the uridine–adenosine pair because the fluorine atom is more hydrophobic than uridine and cannot serve as a hydrogen bond acceptor.

Zhang and colleagues^88^ reported that a 5-nitroindole modification at position 15 of the siRNA passenger strand greatly reduced the activity of the passenger strand, whereas the effectiveness of the siRNA guide strand was barely affected. This modification provided a practical strategy to reduce the passenger strand-mediated off-target effects.

siRNAs containing 5-fluoro-2′-deoxyuridine (FdU) moieties can suppress targeted gene expression. The effects depend on the locations of the FdU substitution. In addition, these modified siRNAs quickly release FdU after entering the cell, thereby further inducing a variety of DNA-damage repair and apoptosis pathways and ultimately triggering cell death. These findings provide a strategy for siRNA-based cancer therapy.^89^

Moreover, many other less common base analogs have also been used in siRNA modifications. Their application helps researchers better understand the mechanism of gene silencing and helps develop new methods to mitigate off-target effects caused by harmful protein binding or undesired targeting of mRNA.^90^

### Modification patterns used in clinical studies or for commercial siRNA

Early siRNA therapeutic programs typically employ partial or slight modifications by using 2′-OMe, 2′-F or PS,^91,92^ e.g., alternative 2′-OMe modifications were placed in the antisense strand of QPI-1007, whereas just one L-DNA was used in the sense strand. After making great efforts to understand the effects of various modifications on siRNA activity, specificity and toxicity, researchers have realized that heavy modification of siRNA, even full-length embellishment, probably do not affect siRNA activity. Furthermore, these judicious modification strategies can significantly enhance siRNA stability or biocompatibility. As a result, several different, interrelated modification patterns have been proposed and validated preclinically and clinically.

Standard template chemistry (STC) (Fig. 3) is a universal modification pattern established by Alnylam Pharmaceuticals. When the lengths of sense and antisense siRNA are 21 and 23 nt, respectively (referred to as “S21nt” and “AS23nt”, respectively, in the following description), 2 PS linkages are placed at the 3′ terminus of the antisense strand, three consecutive 2′-F moieties are incorporated at positions 9, 10 and 11 of the sense strand from the 5′-end, and consecutive 2′-OMe modifications are placed at positions 11, 12 and 13 of the antisense strand from the 5′-end. Moreover, alternative 2′-OMe and 2′-F moieties are employed at other positions in both strands. It is worth mentioning that 2′-OMe and 2′-F are complementarily used for all positions of both strands of siRNA (Fig. 3). It is considered that placing three consecutive 2′-F moieties, instead of larger groups, at the middle of the sense strand aims to facilitate the cleavage and removal of the sense strand in RISC, as release of the sense strand is the prerequisite of RISC activation. PS linkages and OMe positioned at the terminals are used to enhance the capability of siRNA to resist degrading enzyme attack.

**Fig. 4:**
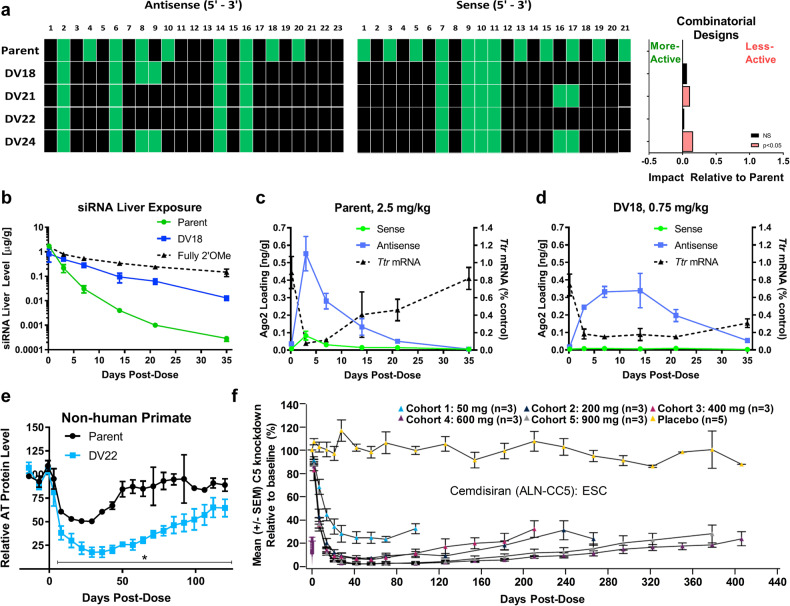
Preclinical and clinical performance of siRNAs with comprehensive modification chemistries. **a** Sense and antisense strand optimization by using transthyretin (Ttr)-targeting siRNAs and the combinatorial designs explored across 10 siRNAs. The effect relative to the parent is described as the model-adjusted mean difference in the activity of the design variant (DV) compared to the parent. **b**–**d** The liver exposure, RISC loading, and silencing activities of the parent, DV18, and fully 2′-OMe conjugates. Data are shown as the mean ± SD. **e** Gene-silencing duration of antithrombin-targeted siRNAs modified with the designs of patent and DV22 evaluated in *Cynomolgus* monkeys (*n* = 3 per group). Asterisks indicate that a significant difference was observed between the parent and DV22. Error is shown as the SD. **f** C5 knockdown profile after applying a single dose of Cemdisiran that employs ESC modification in a phase 1/2 clinical study. Error is represented as the SEM. **a**–**e** Copyright of Elsevier Inc., 2018. **f** Copyright of Alnylam Pharmaceuticals, 2016

The STC design (e.g., as used in revusiran) has been proven to remarkably enhance siRNA stability and affinity without compromising intrinsic RNAi activity.^93^ ALN-AT3, another STC-modified RNAi therapeutic, also showed potent and durable gene silencing after single subcutaneous (s.c.) administration.^94^ However, the clinical investigation of revusiran was discontinued due to unbalanced death between revusiran- and placebo-treated groups. Although the detailed investigation report by Alnylam illustrated that the deaths of revusiran-treated patients were not correlated with the test drug, there are still strong concerns regarding STC-modified siRNA therapeutics. Hence, the next generation of modification patterns, called enhanced stabilization chemistry (ESC) (Fig. 3), was proposed. This design includes four more PS linkages at the 5′-end of the antisense strand and the 3′-end of the sense strand. Another important change was the reduction in 2′-F substitutions, probably because heavy use of 2′-F may magnify toxicity.^95^ These changes markedly enhanced siRNA potency and duration compared with the changes inherent to the STC design.^9,96,97^ This advancement allowed us to achieve the desired pharmacodynamic effect at a significantly lower dose, with a markedly reduced dosing frequency.^98^ Cemdisiran (ALN-CC5) achieved potent gene silencing as long as more than 1 year after receipt of a single dose of the testing therapeutic in a phase 1/2 clinical study (Fig. 4f), representing an unprecedented milestone in pharmaceutical history. Moreover, the newly approved GIVLAARI™ is an siRNA therapeutic based on the ESC design (Fig. 3).^99^

In addition, Alnylam comprehensively optimized the modification design variants (DVs) of the ESC strategy across multiple siRNAs with an iterative screening approach to further enhance stability without compromising intrinsic RNAi activity. As a result, advanced ESC designs, e.g., DV18 and DV22, were proposed (Figs. 3 and 4a–e).^96^ Compared to the former scheme, the advanced strategy maintains six PS linkages at three strand terminals; however, this modification scheme significantly reduces the proportion of 2′-F moieties. Only 10 and 8 2′-F substitutions are used in two strands for the advanced ESC designs of DV18 and DV22, respectively. For DV18, the 2′-F modifications are positioned at sites 7, 9, 10 and 11 in the sense strand and sites 2, 6, 8, 9, 14 and 16 in the antisense strand (all from the 5′-ends of the strands). Compared with DV18, DV22 has the 2′-F modifications at 8 and 9 in the antisense strand further replaced with 2′-OMe (Figs. 3 and 4a). Both the DV18 and DV22 designs can achieve significantly higher liver exposure and RISC loading and more potent and durable gene silencing than the parent design in preclinical species, including nonhuman primates (Fig. 4b–e). The underlying mechanisms were not fully unveiled in Alnylam’s literature. However, Zheng et al.^100^ demonstrated that modification of the 14th position of the siRNA guide strand could eliminate its gene-silencing activity by reducing RISC loading and target degradation, and the larger the modification group used was, the higher the reduction efficiency. Song et al.^28^ further proved that decorations at positions 9 and 10 (for siRNA with a 19 nt/19 nt structure) markedly reduced the activity of the unmodified strand of siRNA without disturbing the potency of the modified strand. In light of this, the specificity and activity of siRNAs could be improved by introducing 2′-MOE at the cleavage site of the siRNA. These observations are in line with the rationales of advanced ESC designs.

Furthermore, investigators from Alnylam have disclosed that the hepatotoxicity of GalNAc (*N*-acetylgalactosamine)-siRNA conjugates is attributed to off-target gene silencing mediated by miRNA-like recognition between siRNA and a mistargeted RNA.^31^ Researchers have demonstrated that disorganizing the nucleotides of the seed region without changing the 2′-OMe, 2′-F or PS content or placing a GNA at position 7 of the siRNA antisense strand can markedly alleviate off-target effects and mitigate hepatotoxicity because these modifications affect the binding of siRNA with undesired target mRNA in a seed region-specific manner. The employment of GNA in the siRNA seed region is the main technical characteristic for the ESC+ design compared to advanced ESC designs (Fig. 3). Several investigational siRNA therapeutics of Alnylam, e.g., ALN-AAT02, ALN-HBV02, and ALN-AGT, utilize the ESC+ design as the modification pattern.

In addition to Alnylam, Arrowhead Pharmaceuticals (Arrowhead) proposed a series of new modification designs (Fig. 3) by combining the modification patterns (e.g., inverted bases) of siRNAs with 2′-OMe/2′-F decorations similar to the aforementioned STC and ESC modification patterns. Typically, the modification strategies proposed by Arrowhead are characterized by placing inverted deoxythymine (idT) at the strand terminus and/or termini, including UNA and X (without nucleoside base), flanking the UAU or UAUAU motif(s), conjugating the siRNA with cholesterol or other hydrophobic substrates, etc. These designs are currently being investigated in clinical trials.^101^ Dicerna Pharmaceuticals (Dicerna)^102^ employed their proprietary dicer-substrate siRNA (DsiRNA) technology to develop RNAi therapeutics. In addition, these researchers also established a series of DsiRNA modification chemistries. For instance, three consecutive 2′-F moieties may be placed at sites 9, 10, and 11 in the sense strand of siRNA from the 5′-end, and the other sites may use alternative 2′-OMe and 2′-F moieties in the flank sequence. One example of a constant flank sequence is ‘GCAGCCGAAAGGUGC’, which contains inner complementary pairing motifs of ‘GCAGCC’ and ‘GGCUGC’. Consecutive 2′-OMe moieties may be used to modify these motifs, and consecutive RNA or DNA nucleosides without additional 2′ modifications may be employed for the bubble motif of GAAA. Moreover, DNA nucleosides can also be applied at sites 2, 12, 16, 18, 20 and 21 in the antisense strand from the 5′-end. PS linkages may be incorporated into the antisense strand at specific sites, as well as within the constant flank sequence (Fig. 3). The ligands may be placed at the bubble sequence. Silence Therapeutics^103^ (Fig. 3) and other RNAi-based biotech companies, such as Arbutus Biopharma, OliX Pharmaceuticals and Suzhou Ribo Life Science, have all been devoted to developing state-of-the-art siRNA modification chemistries to establish fruitful drug development pipelines.^2^

## siRNA delivery and drug development

### Barriers to siRNA delivery

RNAi modalities (siRNAs and miRNAs) need to employ proteins (such as Argonaute and Tar RNA binding protein) in the cytoplasm. A series of barriers need to be circumvented for systemically administered exogenous siRNA before it can achieve gene silencing,^104–106^ such as nuclease degradation and short-lived circulation, immune recognition in blood circulation, accumulation in desired tissue, effective transmembrane trafficking, and escape from endosomes and lysosomes to the cytoplasm. By incorporating chemical modifications, the challenges of stability against serum nucleases and avoiding immune recognition have been substantially reduced. However, other problems remain to be solved.

In blood circulation, unspecific binding and glomerular filtration both hamper the accumulation of siRNA in desired tissues. The neutral surface charge of siRNA-loaded nanoformulations is beneficial for avoiding unfavorable protein binding in circulation.^107^ However, protein decoration or protein corona formation is required for nanoformulation and attachment to specific tissues or cells for some delivery systems.^108,109^ For instance, ionizable lipid nanoparticles (iLNPs) primarily accumulate in the liver by interacting with serum lipoproteins, e.g., apolipoprotein E3 (ApoE3), which leads to specific recognition by the receptor, e.g., low-density lipoprotein receptor (LDLR).^108,109^ In contrast, ligand–siRNA conjugates can transport siRNA to desired tissues and cells by specific recognition and interactions between the ligand, e.g., a carbohydrate, peptide, antibody, aptamer, small molecule, etc., and the surface receptor. This active targeting strategy not only reduces siRNA accumulation in unintended tissues, thus decreasing or avoiding undesired side effects and toxicity, but also achieves potent gene silencing at a low dosage.^104^

**Fig. 5:**
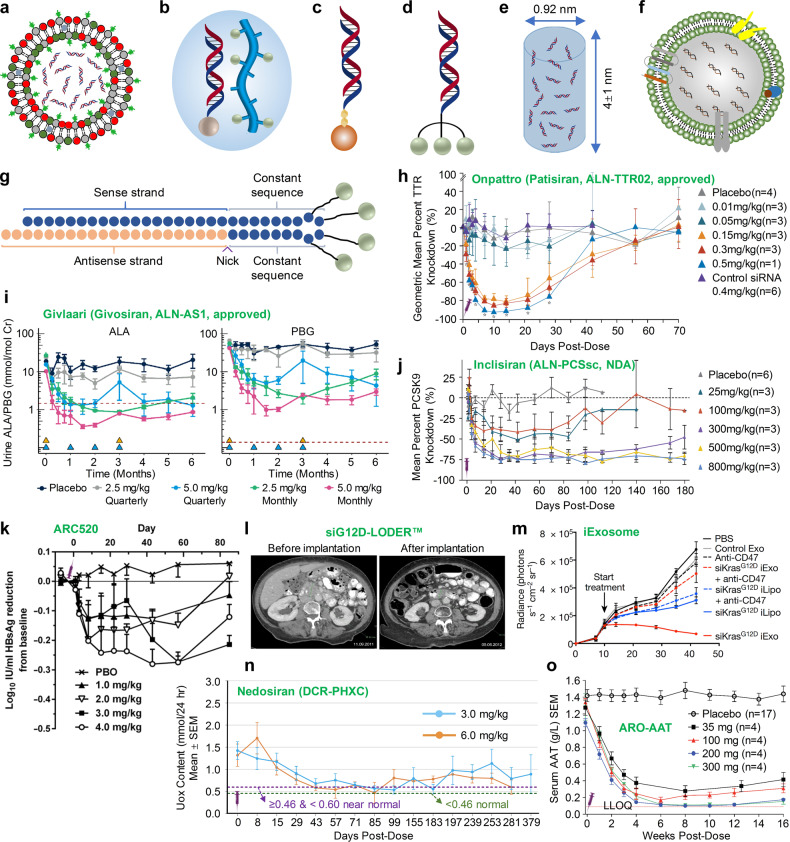
Schemes of representative clinically studied siRNA formulations and their pharmacodynamic performance. **a**–**g** Schemes of **a** lipid nanoparticles, **b** DPC™ or EX-1™, **c** TRiM™, **d** GalNAc–siRNA conjugates, **e** LODER™, **f** iExososme and **g** GalXC™. **h** Efficacy of ONPATTRO^®^ (a liposome formulation) in healthy volunteers, in which siRNA targeting PCSK9 is formulated in lipid nanoparticles.^34^ Copyright Massachusetts Medical Society, 2013. **i** Levels of urinary aminolevulinic acid (ALA) and porphobilinogen (PBG) after once-monthly and once quarterly doses of GIVLAARI™ in acute hepatic porphyria (AHP) patients. Copyright Alnylam Pharmaceuticals, 2019. **j** Reduction of plasma PCSK9 after a single increasing dose of inclisiran.^38^ Copyright Massachusetts Medical Society, 2017. **k** Serum HBsAg reduction in hepatitis B patients who received the treatment of a single dose of ARC-520 (1–4 mg/kg) (formulated with DPC2.0) on a background of daily oral NUCs.^264^ PBO, patients on NUC therapy receiving placebo injection. NUCs nucleos(t)ide viral replication inhibitors. The error is shown as the SEM. Copyright American Association for the Advancement of Science, 2017. **l** Antitumor effect of siG12D-LODER™ combined with chemotherapy in locally advanced inoperable pancreatic cancer in a patient. A computed tomography (CT) scan was performed before and after (nine months later) the administration of siG12D-LODER™. The tumor measured 35.42 and 26.16 mm in the longest diameter, respectively. **m** PANC-1 tumor growth in animals receiving iExosomes or other control formulations.^238^*N* = 3 mice per group. Copyright Macmillan Publishers Limited, part of Springer Nature, 2017. **n** The 24-h Uox (urinary oxalate) values of healthy volunteers and patients treated with a single dose of Nedosiran (DCR-PHXC, a GalXC™ therapeutic) (3 and 6 mg/kg).^265^ Copyright Dicerna Pharmaceuticals, 2020. **o** Reduction of serum AAT in volunteers receiving a single dose of ARO-AAT (a TRiM™ therapeutic, 35–300 mg).^266^ The error bars show the SEM. Copyright Arrowhead Pharmaceuticals, 2019

**Fig. 6:**
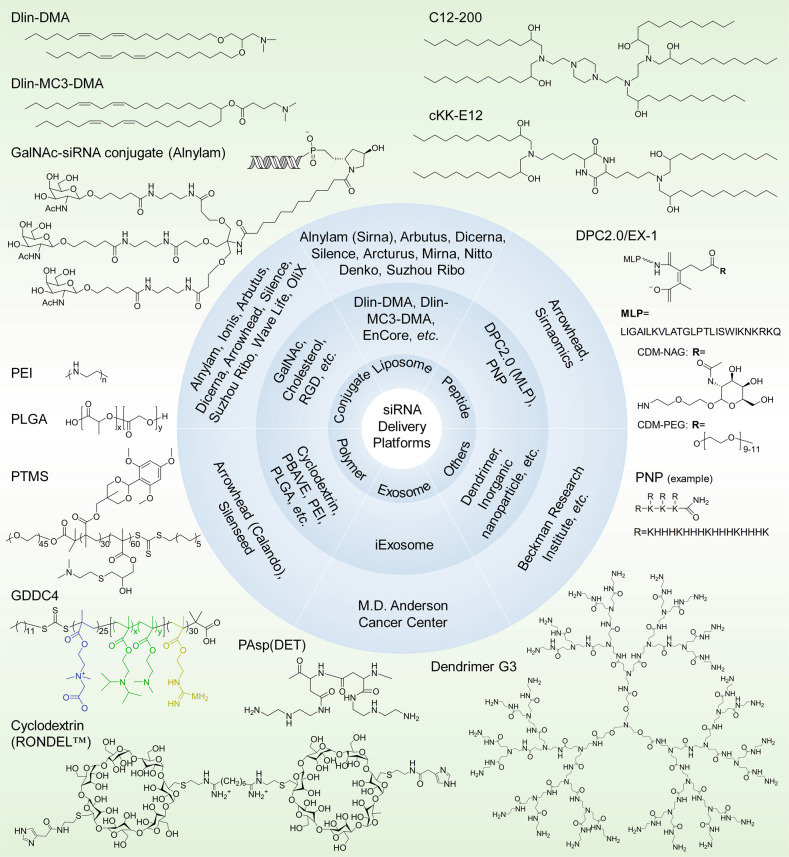
siRNA delivery platforms that have been evaluated preclinically and clinically. Varieties of lipids or lipidoids, siRNA conjugates, peptides, polymers, exosomes, dendrimers, etc. have been explored and employed for siRNA therapeutic development by biotech companies or institutes. The chemical structures of the key component(s) of the discussed delivery platforms, including Dlin-DMA, Dlin-MC3-DMA, C12-200, cKK-E12, GalNAc–siRNA conjugates, MLP-based DPC2.0 (EX-1), PNP, PEI, PLGA-based LODER, PTMS, GDDC4, PAsp(DET), cyclodextrin-based RONDEL™ and dendrimer generation 3 are shown. DLin-DMA (1,2-dilinoleyloxy-3-dimethylaminopropane), DLin-MC3-DMA (6Z,9Z,28Z,31Z)-heptatriaconta-6,9,28,31-tetraen-19-yl-4-(dimethylamino) butanoate, DPC Dynamic PolyConjugates, MLP membrane-lytic peptide, CDM carboxylated dimethyl maleic acid, PEG polyethylene glycol, NAG *N*-acetylgalactosamine, PNP polypeptide nanoparticle, PEI poly(ethyleneimine), LODER LOcal Drug EluteR, PLGA poly(lactic-co-glycolic) acid, PTMS PEG-PTTMA-P(GMA-S-DMA) poly(ethylene glycol)-co-poly[(2,4,6-trimethoxybenzylidene-1,1,1-tris(hydroxymethyl))] ethane methacrylate-co-poly(dimethylamino glycidyl methacrylate), GDDC4 PG-P(DPAx-co-DMAEMAy)-PCB, where PG is guanidinated poly(aminoethyl methacrylate) PCB is poly(carboxybetaine) and P(DPAx-co-DMAEMAy) is poly(dimethylaminoethyl methacrylate-co-diisopropylethyl methacrylate), PEG-PAsp(DET) polyethylene glycol-b-poly(*N*′-(*N*-(2-aminoethyl)-2-aminoethyl) aspartamide), PBAVE polymer composed of butyl and amino vinyl ether, RONDEL™ RNAi/oligonucleotide nanoparticle delivery

siRNA is ~7–8 nm in length and 2–3 nm in diameter. This molecule is too large to cross cell membranes but small enough to be freely cleared by glomeruli,^110^ as molecules with a size smaller than 8 nm^111^ are easily filtered into the urine. Hence, once siRNAs leave the bloodstream, they will accumulate in the bladder and be excreted out from the body quickly, within a few minutes to half an hour,^48–50^ which prevents them from accumulating in targeted tissues or cells. Encapsulating siRNA into vesicles or conjugating it to certain ligands can effectively avoid renal clearance and, more importantly, can deliver siRNA to the desired tissues or cells. Lipid nanoparticles (LNPs),^106^ dynamic polyconjugates (DPC™), and GalNAc–siRNA conjugates have all achieved efficient siRNA delivery to hepatocytes (Figs. 5, 6).^112^

The relatively high molecular weight (~13–16 kD) and net negative charge prevent artificial siRNA from crossing the cell membrane. Hence, we attempted to determine whether any cell can internalize siRNA without a carrier, leading to the conclusion that naked siRNA can only be taken up by a few cell types, e.g., retinal ganglion cells (RGCs) and neurons. In addition, researchers have also been devoted to identifying various carriers to achieve efficient transmembrane delivery. As a result, cationic cell-penetrating peptides (CPPs) become a choice at the early stage. CPPs, typically tailed with arginine-rich sequences, can form bidentate bonds by the interaction between the guanidinium groups and the negative phosphates, sulfates and carboxylates on the cell surface.^113^ This interaction causes membrane pore formation, leading to the cellular uptake of siRNA. As another strategy, the negative charge of siRNA can be neutralized by positively charged lipids or polymers, conferring siRNA the ability to more readily bind to the membrane and become easily internalized via adsorptive pinocytosis.

Finally, siRNAs must effectively escape from endosomes and lysosomes to the cytoplasm, where antisense strands of siRNAs need to be loaded into RISCs. Many delivery systems employ a pH-sensitive unit to respond to pH changes in the endosome and lysosome,^114,115^ where they will absorb H^+^, presenting a positive charge on the surface. Then, the osmotic pressure will increase in the endosome or lysosome, resulting in the internal flow of Cl^−^ and H_2_O. Finally, these changes may cause membrane disruption and siRNA release to the cytoplasm. This so-called ‘proton sponge effect’ or ‘colloid osmotic pressure effect’ results in membrane destabilization^116–119^ or membrane swelling,^120,121^ respectively. However, the underlying mechanism of endosomal release remains to be further illuminated. Only 1–2% of internalized LNP-loaded siRNAs were released into the cytoplasm, and this only occurred within a limited time frame after internalization.^122,123^ Hence, further understanding the escape mechanism and how to enhance the escape efficiency is of great importance for siRNA drug development. Recently, Wang and colleagues^124^ developed novel endoplasmic reticulum (ER) membrane-modified hybrid nanoplexes (EhCv/siRNA NPs). Compared with unmodified nanoplexes, they showed much higher RNAi activity in vitro and in vivo. The functional proteins on the ER membrane have an important role in intracellular trafficking of siRNA, helping siRNA reach the cytoplasm through the endosome–Golgi–ER pathway instead of the endosome–lysosome pathway, thereby avoiding the lysosomal degradation of siRNA. In addition, electroporation enables siRNA to directly cross the cell membrane, which also constitutes an effective approach to circumvent the endosomal escape issue.^125–131^

To date, two RNAi therapeutics, ONPATTRO^®^ and GIVLAARI™, have been approved for commercial application, and two siRNAs, lumasiran (ALN-GO1) and inclisiran, have been submitted for new drug application (NDA) to the FDA. Seven siRNAs are undergoing phase 3 clinical studies, and more candidates are in the early developmental stage. Various delivery systems, e.g., LNPs, DPC™, TRiM™, GalNAc–siRNA conjugates, LODER™ polymers, exosomes and polypeptide nanoparticles (PNPs), have been explored (Figs. 5, 6). Based on these systems, plentiful drug pipelines have been established. We discuss this information in the following sections.

### Naked siRNA-based therapeutics

Naked siRNA can be defined as a system that contains no delivery system that is associated with siRNA either covalently or noncovalently.^40^ Because no protective effect is offered by the delivery vehicle, siRNA should be modified carefully to be resistant to enzyme degradation and extend its circulation time in the bloodstream. As siRNA is naturally filtered to the kidney, it can be used to silence kidney-expressed genes and treat renal diseases.^132^ Alternatively, a feasible strategy for naked siRNA drug development is the local injection of this molecule into specific organs that are relatively closed off and contain few nucleases, e.g., the eye.

QPI-1002 (I5NP)^132,133^ and QPI-1007^36,134^ were developed by Quark Pharmaceuticals (Table 1). QPI-1002 is a 19-base pair, 2′-O-methylated, blunt and naked siRNA^135^ targeting p53 for treating acute kidney injury (phase 2) and delayed graft function (phase 3). The sequence of the sense strand is 5′-GAAGAAAATTTCCGCAAAA-3′.^135^ Because of their small sizes, most siRNAs accumulate in the kidney after being intravenously administered, achieving concentrations 40 times greater than those in other organs, followed by rapid entry into proximal tubule cells. An in vitro study determined that QPI-1002 elicited near-complete p53 mRNA elimination at a concentration of ~1 nM, with an IC50 of ~0.23 nM. This molecule also effectively inhibited p53 protein expression, even at a transfection concentration of 0.5 nM. Furthermore, bilateral renal-clamp studies were conducted to identify the effect of siRNA on the preservation of kidney function. Compared with PBS-treated animals, siRNA-treated animals showed ischemia with decreased serum creatinine levels from 3.7 mg/dL to 1.9 mg/dL. Adverse effects (AEs) were found only when the dose was higher than 1000 mg/kg in nonhuman primates and higher than 800 mg/kg in rats.^133^ A phase 1 clinical study (NCT00554359) of QPI-1002 revealed an ideal safety profile.^36,132^

**Table 1:** Clinical and preclinical activities of RNAi therapeutics: selected examples

Therapeutic name	Condition(s)	Modification chemistry	Delivery system	Target(s)	Sponsor	Phase	NCT ID	References
ONPATTRO® (patisiran, ALN-TTR02)	TTR-mediated amyloidosis	2′-OMe, 2′-F	LNP (DLin-MC3-DMA)	TTR	Alnylam Pharmaceuticals	Approved, 3	NCT03862807 NCT03997383 NCT03759379 NCT02510261 NCT01617967 NCT01559077 NCT02939820 NCT01961921 NCT01960348 NCT02053454 NCT03431896	^35,180^
GIVLAARI™ (givosiran, ALN-AS1)	Acute hepatic porphyrias	PS, 2′-OMe, 2′-F	GalNAc–siRNA conjugate	ALAS1	Alnylam Pharmaceuticals	Approved, 3	NCT02452372 NCT02949830 NCT03505853 NCT03338816 NCT04056481	^9,10^
Lumasiran (ALN-GO1)	Primary hyperoxaluria type 1	PS, 2′-OMe, 2′-F	GalNAc–siRNA conjugate	HAO1	Alnylam Pharmaceuticals	3	NCT03905694 NCT03681184 NCT02706886 NCT03350451	^267^
Vutrisiran (ALN-TTRSC02)	Amyloidosis	PS, 2′-OMe, 2′-F	GalNAc–siRNA conjugate	TTR	Alnylam Pharmaceuticals	3	NCT03759379	^98^
Inclisiran (ALN-PCSsc)	Hypercholesterolemia	PS, 2′-OMe, 2′-F	GalNAc–siRNA conjugate	PCSK9	Alnylam Pharmaceuticals	3	NCT03814187 NCT03851705 NCT03705234 NCT03159416 NCT03060577 NCT03399370 NCT03400800 NCT03397121 NCT02314442 NCT02597127 NCT01437059 NCT02963311	^38^
Fitusiran (ALN-AT3SC)	Hemophilia	PS, 2′-OMe, 2′-F	GalNAc–siRNA conjugate	AT	Alnylam Pharmaceuticals partnered with Genzyme	3	NCT03549871 NCT03974113 NCT03754790 NCT03417102 NCT03417245	^213^
Cemdisiran (ALN-CC5)	Complement-mediated diseases	PS, 2′-OMe, 2′-F	GalNAc–siRNA conjugate	C5	Alnylam Pharmaceuticals	2	NCT03999840 NCT03841448	^268,269^
ALN-AAT02	Alpha-1 liver disease	PS, 2′-OMe, 2′-F	GalNAc–siRNA conjugate	AAT	Alnylam Pharmaceuticals	1/2	NCT03767829	^269^
ALN-AGT	Hypertension	PS, 2′-OMe, 2′-F, GNA	GalNAc–siRNA conjugate	AGT	Alnylam Pharmaceuticals	1	NCT03934307	^269–271^
ARO-AAT	Alpha-1 antitrypsin deficiency	PS, 2′-OMe, 2′-F, inverted base	GalNAc–siRNA conjugate	AAT	Arrowhead Pharmaceuticals	2/3	NCT03946449 NCT03362242 NCT03945292	^272^
ARO-HBV	Hepatitis B	PS, 2′-OMe, 2′-F, inverted base	GalNAc–siRNA conjugate	HBV gene	Arrowhead partnered with Janssen	1/2	NCT03365947	^273^
ARO-APOC3	Hypertriglyceridemia, familial chylomicronemia	PS, 2′-OMe, 2′-F, inverted base	GalNAc–siRNA conjugate	ApoC3	Arrowhead Pharmaceuticals	1	NCT03783377	^274^
ARO-ANG3	Hypertriglyceridemia	PS, 2′-OMe, 2′-F, inverted base	GalNAc–siRNA conjugate	ANGPTL3	Arrowhead Pharmaceuticals	1	NCT03747224	^275^
AMG 890	Cardiovascular disease	Undisclosed	GalNAc–siRNA conjugate	Lp(a)	Arrowhead Pharmaceuticals partnered with Amgen	2	NCT03626662	^276,277^
ND-L02-s0201	Idiopathic pulmonary fibrosis	Undisclosed	LNP, vitamin A	HSP47	Bristol-Myers Squibb	2	NCT03538301 NCT03241264 NCT02227459 NCT01858935	^159,278^
DCR-PHXC	Primary hyperoxaluria	Undisclosed	GalNAc–siRNA conjugate	LDHA	Dicerna Pharmaceuticals	3	NCT03847909 NCT03392896	^218,219^
DCR-HBVS	Hepatitis B	Undisclosed	GalNAc–siRNA conjugate	HBV gene	Dicerna Pharmaceuticals	1	NCT03772249	^14,219^
SV40 vectors carrying siRNA	Bcr-Abl	Undisclosed	Pseudoviral (SV40) particles	Chronic myeloid leukemia	Hadassah Medical Organization	NS	NCT00257647	^279^
BMT101	Hypertrophic scar	Undisclosed	cp-asiRNA	CTGF	Hugel	2	NCT04012099 NCT03133130	
SXL01	Advanced cancers	Undisclosed	NA	AR	Institut Claudius Regaud	1	NCT02866916	
Mesenchymal stromal cell-derived exosomes with KRAS-G12D-targeting siRNA	Pancreatic cancer	Unknown	Exosome	Kras G12D Mutation	M.D. Anderson Cancer Center	1	NCT03608631	^238^
siRNA-EphA2-DOPC	Advanced cancers	Undisclosed	Liposome	EphA2	M.D. Anderson Cancer Center	1	NCT01591356	^280–282^
NU-0129	Gliosarcoma	Undisclosed	Gold nanoparticle	BCL2L12	Northwestern University	1	NCT03020017	^283^
OLX10010	Hypertrophic cicatrix	2′-OMe, PS	cp-asiRNA (cholesterol-siRNA conjugate)	CTGF	Olix Pharmaceuticals	1	NCT03569267	^284^
TD101	Pachyonychia congenita	Undisclosed	None	Keratin 6A N171K mutant	Pachyonychia Congenita Project	1	NCT00716014	^285^
QPI-1002 (I5NP)	Delayed graft function, other complication of kidney transplant	2′-OMe	None	p53	Quark Pharmaceuticals	3	NCT03510897 NCT02610296 NCT02610283 NCT00802347	^132^
QPI-1007	Nonarteritic anterior ischemic optic neuropathy	2′-OMe	None	Caspase-2	Quark Pharmaceuticals	3	NCT01965106 NCT01064505	^134^
PF-655 (PF-04523655)	Choroidal neovascularization, diabetic retinopathy, diabetic macular edema	2′-OMe	None	RTP801	Quark Pharmaceuticals	2	NCT01445899	^286^
PSCT19 (MiHA-loaded PD-L-silenced DC caccination)	Hematological malignancies	Undisclosed	Ex vivo transfection	PD-L1/L2	Radboud University	1/2	NCT02528682	^287,288^
RXI-109 (sd-rxRNA)	Hypertrophic scar	Undisclosed	None	CTGF	RXi Pharmaceuticals	2	NCT02599064 NCT02246465 NCT01780077 NCT01640912 NCT02079168 NCT02030275	^289^
Atu027	Pancreatic ductal carcinoma, advanced solid tumors	2′-OMe	AtuPlex	PKN3	Silence Therapeutics GmbH	1/2	NCT01808638 NCT00938574	^157,175,290^
SLN124	Nontransfusion-dependent thalassemia low-risk myelodysplastic syndrome	Undisclosed	GalNAc–siRNA conjugate	TMPRSS6	Silence Therapeutics plc	1	NCT04176653	
siG12D-LODER	Pancreatic ductal adenocarcinoma, pancreatic cancer	Undisclosed	Polymeric matrix	KRAS G12D	Silenseed Ltd	2	NCT01676259 NCT01188785	^291^
STP705 (cotsiranib)	Hypertrophic scar	Undisclosed	HKP	TGF-β1 and Cox-2	Sirnaomics	1/2	NCT02956317	^237^
STP705	Bowen’s disease cutaneous squamous cell carcinoma in situ	Undisclosed	Histidine-lysine co-polymer (HKP) peptide	TGF-β1 and COX-2	Sirnaomics	1/2	NCT04293679	
Tivanisiran (SYL1001)	Dry eye disease, ocular pain	Undisclosed	None	TRPV1	Sylentis, S.A	3	NCT03108664 NCT01438281 NCT01776658 NCT02455999	^292,293^
Bamosiran (SYL040012)	Ocular hypertension, glaucoma	Undisclosed	None	ADRB2	Sylentis, S.A	2	NCT01227291 NCT00990743 NCT01739244 NCT02250612	^294,295^
APN401 (siRNA-transfected PBMCs)	Solid tumors that are metastatic or cannot be removed by surgery	Undisclosed	Ex vivo siRNA electroporated PBMCs	Cbl-b/DC	Wake Forest University Health Sciences	1	NCT03087591 NCT02166255	^296,297^
Cobomarsen (MRG-106)	Blood cancers (cutaneous T cell lymphoma, adult T cell lymphoma/leukemia, diffuse large B cell lymphoma, chronic lymphocytic leukemia, mycosis fungoides)	LNA (antimiR)	NA	MicroRNA-155	miRagen Therapeutics	2	NCT02580552 NCT03713320 NCT03837457	^298^
Remlarsen (MRG-201)	Pathologic fibrosis (cutaneous fibrosis, idiopathic pulmonary fibrosis, keloid, etc.)	2′-OMe, 2′-F, mismatch, PS, Chol (microRNA-29b mimic)	NA	CTGF	miRagen Therapeutics	2	NCT03601052 NCT02603224	^299,300^
MRG-110 (S95010)	Ischemic conditions (heart failure, incisional complications, wound healing)	LNA (antimiR)	NA	MicroRNA-92	miRagen Therapeutics	1	NCT03603431	^301^
ALN-HBV02 (VIR-2218)	Hepatitis B	PS, 2′-OMe, 2′-F,GNA	GalNAc–siRNA conjugate (ESC)	HBV gene	Alnylam Pharmaceuticals	1/2	NCT03672188	^302^
ARO-HIF-2	Clear cell renal cell carcinoma (ccRCC)	PS, 2′-OMe, 2′-F,iB	TRiM (RGD-siRNA conjugate)	HIF-2α	Arrowhead Pharmaceuticals	1	NCT04169711	
ALN-APP	Cerebral amyloid angiopathy	PS, 2′-OMe, 2′-F	Undisclosed	APP	Alnylam pharmaceuticals	Preclinical	N/A	
ARO-ENaC	Cystic fibrosis	PS, 2′-OMe, 2′-F,iB	TRiM (EpL-siRNA conjugate)	αENaC	Arrowhead Pharmaceuticals	Preclinical	N/A	
ARO-AMG1	Undisclosed	PS, 2′-OMe, 2′-F,iB	TRiM	Undisclosed	Arrowhead Pharmaceuticals	Preclinical	N/A	
SLN360	Cardiovascular disease	Undisclosed	GalNAc–siRNA	Lp(a)	Silence Therapeutics	Preclinical	N/A	
SLN226	Alcohol use disorders	Undisclosed	Conjugate	ALDH2	Silence Therapeutics	Preclinical	N/A	
AB-729	Hepatitis B	Undisclosed	GalNAc–siRNA	HBV gene	Arbutus Biopharma Corporation	Preclinical	N/A	
si-PT-LODER	Prostate cancer	Undisclosed	Polymeric matrix (LODER polymer)	HSP90	Silenseed Ltd	Preclinical	N/A	
RBD1016	Hepatitis B	Undisclosed	GalNAc–siRNA conjugate	HBV gene	Suzhou Ribo Life Science Co., Ltd	Preclinical	N/A	
RB-HLP002	Hyperlipidemia	Undisclosed	GalNAc–siRNA conjugate	Undisclosed	Suzhou Ribo Life Science Co., Ltd	Preclinical	N/A	
SYL116011	Allergic conjunctivitis (ophthalmology)	Undisclosed	Naked siRNA	Orai1	Sylentis, S.A.	Preclinical	N/A	
SYL1801	Choroidal neovascularization (CNV)	Undisclosed	Naked siRNA	NRARP	Sylentis, S.A.	Preclinical	N/A	
DCR-BCAT	Cancer	Undisclosed	EnCore Lipid Nanoparticle	CTNNB1	Dicerna Pharmaceuticals, Inc.	Preclinical	N/A	
DCR-AATsc	Antitrypsin deficiency, liver disease	Undisclosed	GalNAc-DsiRNAEX conjugate	AAT	Dicerna Pharmaceuticals, Inc.	Preclinical	N/A	
OLX301A	Age-related macular degeneration/retinal fibrosis	Undisclosed	cp-asiRNA	CTGF	OliX pharmaceuticals, Thea	Preclinical	N/A	

*2*′-*F* 2′-fluoro substitution, *2*′-*OMe* 2′-methoxy group substitution, *LNA* locked nucleic acid, *LNP* lipid nanoparticle, *DLin-MC3-DMA* (6Z,9Z,28Z,31Z)-heptatriaconta-6,9,28,31-tetraen-19-yl-4-(dimethylamino)butanoate, *TTR* transthyretin, *PS* phosphorothioate linkage, *GalNAcN*-acetyl-d-galactosamine, *HAO1* hydroxyacid oxidase 1, *ALAS1* delta-aminolevulinate synthase 1, *PCSK9* proprotein convertase subtilisin/kexin type 9, *AT* antithrombin, *C5* complement component 5, *AAT* alpha-1 antitrypsin, *AGT* angiotensinogen, *cp-asiRNA* asymmetric siRNA, *HBV* hepatitis B virus, *ApoC3* apolipoprotein C3, *ANGPTL3* angiopoietin-like 3, *Lp(a)* lipoprotein (a), *HSP47* heat shock protein 47, *LDHA* lactate dehydrogenase A, *CTGF* connective tissue growth factor, *AR* androgen receptor, *EphA2* EPH receptor A2 (ephrin type-A receptor 2), *DOPC* 1,2-dioleoyl-*sn*-glycero-3-phosphocholine, *BCL2L12* B cell lymphoma 2-like protein 12, *RTP801 (Ddit4)* DNA-damage-inducible transcript 4, *PD-L1* programmed death-ligand 1, *PD-L2* programmed death-ligand 2, *PKN3* protein kinase N3, *HKP* histidine-lysine co-polymer, *TRPV1* transient receptor potential cation channel subfamily V member 1, *ADRB2* β2 adrenergic receptor, *PBMC* peripheral blood mononuclear cell, *TGF-β1* transforming growth factor beta 1, *Cox-2* cyclooxygenase-2, *Cbl-b* casitas-B-lineage lymphoma protein-b, *DC* dendritic cell, *APP* amyloid precursor protein, *ENaC* epithelial sodium channel alpha subunit, *HIF-2α* hypoxia-inducible factor-2α, *ALDH* aldehyde dehydrogenase, *HSP90* heat shock protein 90, *Orai1* ORAI calcium release-activated calcium modulator 1, *NRARP* NOTCH-regulated ankyrin repeat protein, *CTNNB1* catenin beta-1 (β-catenin), *NS* not specified, *N/A* not available

QPI-1007 is a caspase-2-targeted siRNA without formulation that was developed to treat acute primary angle closure glaucoma (phase 2) and nonarteritic anterior ischemic optic neuropathy (NAION, phase 3). This molecule is administered via intravitreal injection. It is modified by 2′-OMe in the antisense strand, an l-DNA cytidine nucleotide is located before the last nucleotide at the 3′-end, and the sense strand has an inverted deoxyabasic residue at the 5′-end^36^ (Fig. 3).

Preclinical investigations have validated the potent gene silencing of QPI-1007 in cells and have demonstrated its curative effects on optic nerve-damaged animal models. Data^136^ have shown that QPI-1007 triggered over 80% gene suppression in HeLa cells and exhibited an IC50 of ~0.8 nM against human caspase-2 mRNA. In animal models, eyes treated with siRNA showed a dose-dependent increase in RGC survival from 5 to 20 μg. In particular, in animals dosed with 20 and 35 μg QPI-1007, RGC densities in the injured eye recovered to close to healthy levels (~98%). Furthermore, a phase 1/2a clinical trial (NCT01064505) reported that most common AEs, such as conjunctival hemorrhage, conjunctival edema, eye irritation, and eye pain, were typical of intravitreal injection, and no serious adverse effects (SAEs) were observed. By comparing the best-corrected visual acuity (BCVA) following a single dose of QPI-1007 with natural history historical controls from the Ischemic Optic Neuropathy Decompression Trial (IONDT, 1998), which is the most comprehensive survey of the disease and serves as the gold standard in the field, it was concluded that QPI-1007 significantly protected the optic nerve as the proportions of subjects who lost 3 lines of visual acuity were markedly decreased. The proportions were 0%, 0% and 3.6% at months 3, 6 and 12, respectively, following a single dose of QPI-1007. In contrast, the proportions for natural history controls were 9%, 15% and 16% at months 3, 6 and 12, respectively. Currently, an international multi-centered phase 2b/3 clinical trial is being conducted in the United States, China, Israel and other countries and regions.

ALN-RSV01 (asvasiran sodium), a naked siRNA that targets the respiratory syncytial virus (RSV) nucleocapsid (N) gene and inhibits viral replication, was explored for the potential treatment or prevention of RSV infection. The sequences of ALN-RSV01 are as follows: sense strand (5′–3′): GGCUCUUAGCAAAGUCAAGdTdT; and antisense strand (5′–3′): CUUGACUUUGCUAAGAGCCdTdT.^137^ ALN-RSV01 was administered via inhalation with an investigational electronic nebulizer. Although the clinical study of ALN-RSV01 has been terminated, clinical data indicate that it is well tolerated in vivo^138^ and may have beneficial effects on long-term allograft function in lung transplant patients infected with RSV (NCT00658086).^139,140^ Another clinical result reported in 2016 showed that ALN-RSV01 may help prevent bronchiolitis obliterans syndrome (BOS) after lung syncytial virus infection in lung transplant recipients.^141^

### Lipid and lipidoid-based nanoparticles

#### Representative lipid materials

Lipid or lipidoid nanoparticles (LNPs) (Figs. 5a and 6) were originally developed as vehicles for DNA-based drugs. Subsequently, LNPs have been increasingly used for siRNA delivery because these vesicles can protect entrapped siRNA from nuclease attack and renal clearance and transport siRNA to targeted tissues and cells.^142–144^ Canonical siRNA-LNPs comprise similar components, e.g., cationic or ionizable lipids, DSPC (1,2-distearoyl-*sn*-glycero-3-phosphocholine), and cholesterol and polyethylene glycol (PEG) lipids.^145^ siRNA-LNPs predominantly accumulate in the liver, spleen and kidney after being intravenously injected.

According to the different charge properties of siRNA-binding lipids under neutral conditions, LNPs may be divided into several classes: ionizable LNPs, cationic LNPs and neutral LNPs. Ionizable LNPs are nearly uncharged during circulation but become protonated in a low pH environment, e.g., in the endosomes and lysosomes. These molecules may interact with apolipoprotein E3 (ApoE3), which transports lipids to hepatocytes. Cationic LNPs exhibit a constitutive positive charge in blood circulation and in endosomes or lysosomes. Cationic LNPs also primarily accumulate in hepatocytes; however, this is independent of the ApoE3 interaction. Electrostatic-induced nonspecific binding with plasma protein and relatively higher immunogenicity may cause cationic LNPs to be less efficacious^146^ and more toxic in vivo than ionizable LNPs;^147^ as a consequence, most pharmaceutical firms and institutes have made efforts to develop novel ionizable lipids to achieve efficient hepatocyte-targeted delivery of siRNA with minimal side effects. For example, research teams from Arbutus Biopharma, Alnylam Pharmaceuticals and the Massachusetts Institute of Technology continue to establish lipid-based delivery systems. Three generations of lipid delivery systems have been developed by Arbutus and Alnylam (Fig. 6). They employed DLin-DMA (1,2-dilinoleyloxy-3-dimethylaminopropane),^148^ DLin-MC3-DMA ((6Z,9Z,28Z,31Z)-heptatriaconta-6,9,28,31-tetraen-19-yl-4-(dimethylamino) butanoate)^107^ and L319 (di((Z)-non-2-en-1-yl) 9-((4-(dimethylamino) butanoyl) oxy) heptadecanedioate)^149^ as the key lipids and exhibited median effective doses (ED_50_s) of 1, 0.005 and <0.01 mg/kg, respectively, when loaded with anti-factor VII siRNA and injected via the tail vein. DLin-DMA was used to develop TKM-080301,^150^ ALN-VSP^151^ and ALN-TTR01,^34^ whereas DLin-MC3-DMA was employed in ALN-TTR02^34^ and ALN-PCS^152^ development. L319 was derived from DLin-MC3-DMA by incorporating a biocleavable ester linkage within hydrophobic alkyl chains.^149^ It is readily degraded in vivo, and the metabolite of L319 is a potential substrate for the β-oxidation pathway of fatty acids. L319 is rapidly eliminated from intracellular compartments (plasma, liver and spleen) and is excreted in vivo, which suggests high tolerance of L319-LNPs throughout the body.

Anderson and colleagues^153^ synthesized and screened thousands of lipidoids through a combinatorial chemistry strategy. Consequently, three generations of lipidoid materials were selected from these libraries: 98N12-5(I)-based,^154^ C12-200-based^155^ and cKK-E12-based^153^ LNPs (Fig. 6). In particular, the ED_50_ of LNPs containing cKK-E12 is as low as 0.002 mg/kg, which is the lowest ED_50_ in the liver for all reported LNPs currently.^153^ In addition, many firms, including Dicerna Pharmaceuticals (Dicerna),^156^ Silence Therapeutics,^157^ Sirna Therapeutics,^158^ Nitto Denko Corporation,^159^ Life Technologies^160^ and Suzhou Ribo Life Science,^49,142^ have explored proprietary delivery technologies for preclinical and clinical investigations.

Tumor treatment is another application of LNPs. The discontinuous and fenestrated endothelium in rapidly growing malignant tumors and the poor lymphatic system allow LNPs and internalized siRNAs greater accumulation and slower clearance in solid tumors, referred to as the enhanced permeation and retention (EPR) effect.^161^ The EPR effect has become a mainstay of antitumor nanodrug delivery. However, increasing evidence has shown that LNPs are still primarily enriched in hepatocytes; hence, it is difficult to achieve complete remission merely based on the EPR effect for cancer treatment. An advanced design adds active tumor-targeting agents on the surfaces of LNPs that can target (i) the tumor cell surface, (ii) the tumor extracellular matrix and (iii) the endothelial cell surface receptors of tumor vessels by biological or chemical means.^162^ Tumor-targeting moieties can be selected from antibodies or proteins (e.g., transferrin^163,164^), peptides (e.g., RGD^165^ or octreotide^166^), aptamers or small molecules (e.g., hyaluronan^167–169^ or folate^170^). These ligands, to some extent, facilitate siRNA-loaded LNP accumulation in tumor tissues.

After more than 10 years of technological evolution, some theories have been established to guide the rational design of novel artificial lipids. Manoharan et al.^107^ showed that the p*K*a of lipids or lipidoids is of great importance for delivering siRNA to hepatocytes in vivo. In addition, these researchers elucidated the influences of the PEG-lipid content of siRNA-LNPs on delivery, concluding that PEG-C14 showed the highest delivery efficiency compared with PEG-C16 and PEG-C18, and the incorporation of 1.5 mol% PEG-lipid exhibited ideal potency, as higher proportions of PEG-lipid may decrease the potency.^171^ Moreover, Anderson and colleagues^172^ identified four essential structural and p*K*a criteria for the design of degradable lipidoids: (i) tertiary amine, (ii) O13 tail, (iii) more than 2 hydrophobic tails and (iv) p*K*a ≥ 5.5. This finding is of importance for designing and developing liver-targeted delivery systems based on lipid-like materials.

In addition, Arcturus Therapeutics,^173^ Dicerna Pharmaceuticals,^174^ Silence Therapeutics,^175,176^ Sirna Therapeutics (acquired by Alnylam from Merck & Co., Inc. in 2014)^177,178^ and Suzhou Ribo Life Science^142^ also made great efforts to develop lipid-based siRNA delivery systems, some of which have been advanced to the clinical stage.

#### Lipid-employed siRNA therapeutics

**Fig. 7:**
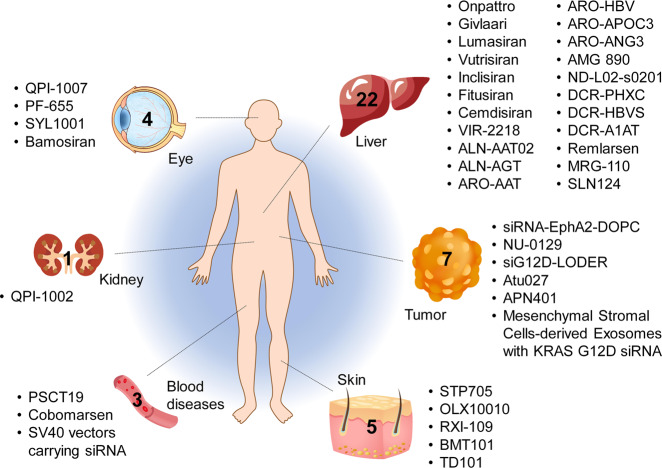
Tissues targeted by siRNA and miRNA therapeutics currently being investigated at the clinical stage. The corresponding therapeutic names are shown beside the tissues

ONPATTRO^®^^78^ is a chemically modified anti-transthyretin (TTR) siRNA formulated in liposomes (Figs. 5a, h and 7). The lipid components of ONPATTRO^®^ include DLin-MC3-DMA (Fig. 6), DSPC, cholesterol and PEG-DMG ((R)-2,3-bis(octadecyloxy)propyl-1-(methoxy polyethylene glycol 2000) carbamate) at a molar ratio of 50/10/38.5/1.5.^107^ The active pharmaceutical ingredient (API, siRNA) of ONPATTRO^®^ is modified with eleven 2′-OMe (nine in the sense strand and two in the antisense strand) and four 2′-deoxy thymidine (two in the sense strand and two in the antisense strand, all at the 3′-end) moieties. Before ONPATTRO^®^, Alnylam advanced ALN-TTR01 to a phase 1 clinical study (NCT01148953). The API of ALN-TTR01 is the same as that of ONPATTRO^®^, whereas the delivery system for ALN-TTR01 comprises Dlin-DMA,^148,179^ cholesterol, DSPC and PEG-DMG. A phase 1 study of ONPATTRO^®^ (NCT01617967) showed a good dose-dependent pattern for TTR protein reduction.^180^ Overall, this drug was well tolerated by subjects, although pretreatment with antihistamines, nonsteroidal antihistamines or glucocorticoids was needed to reduce the incidence of infusion-related reactions. Phase 2 and 3 studies reached both the primary and second endpoints of the studies, according to the outcomes regarding the modified Neuropathy Impairment Score +7 (mNIS+7) and the Norfolk Quality of Life Diabetic Neuropathy (QoL-DN) score. Based on these achievements, the FDA and the EC approved the commercial use of ONPATTRO^®^ in August 2018.

In addition, TKM-080301 (TKM-PLK1) is another DLin-DMA-based LNP formulation.^181^ TKM-080301 was developed by Tekmira Pharmaceuticals and contains anti-PLK1 (polo-like kinase 1) siRNA for the treatment of solid cancer. Preclinical studies have shown ideal antineoplastic activity in xenograft tumor models, and the inhibition of PLK1 expression was maintained at 7–10 days after receipt of a single dose of TKM-080301.^182^ No measurable immune activation or myelosuppressive effects were manifested, and limited toxicity was observed in the liver and spleen. In the phase 1/2 clinical test (NCT02191878), the patients received 28 days of treatment at escalating doses of TKM-080301 from 0.15 to 0.9 mg/kg through a 30-min i.v. infusion once a week. Dose-limiting toxicities (DLTs) and adverse grade 3 reactions were observed in two patients at a dose of 0.9 mg/kg/week, and the maximum tolerated dose (MTD) was 0.75 mg/kg/week.^36,150^ In another clinical trial (NCT01262235), TKM-080301 was dosed at 0.6 or 0.75 mg/kg/week, and the treatment lasted for 18 cycles (4 weeks per cycle). As a result, tumor reduction was achieved, but some serious AEs (SAEs) were also observed.^150^

Moreover, several other RNAi therapeutics also employ liposomes as delivery materials, e.g., ALN-VSP,^151^ ALN-PCS,^152^ MRX34,^183^ Atu027,^175,184^ ARB-1467,^185^ ARB-1740,^186,187^ etc. The clinical studies of all these therapeutics are currently discontinued. ARB-1467 and ARB-1740 are generation 1 and generation 2 anti-HBV modalities, respectively, developed by Arbutus Pharma. ARB-1740 is a clinically investigated and liposome-formulated RNAi agent that contains three siRNAs targeting different regions of the HBV genome.^186,187^ However, the study of ARB-1740 has been suspended because Arbutus transferred their liver-targeted delivery system from liposomes to GalNAc–siRNA conjugates. Accordingly, another anti-HBV siRNA therapeutic, AB-729, is undergoing a phase 1a/1b study,^188^ and positive preliminary clinical results have been disclosed recently.

### DPC™ and TRiM™ delivery platforms

#### Characteristics of DPC™ and TRiM™ delivery platforms

DPC™ or EX-1™^112,189–192^ is a delivery system established by Arrowhead (Fig. 5b). The first-generation DPC (DPC 1.0) technology is characterized by a polymer backbone (polymer composed of butyl and amino vinyl ether, PBAVE) reversibly attached to siRNA, a shielding agent (PEG), and the targeting ligand by a bifunctional maleamate linkage.^112^ In detail, PEG and the targeting ligand are alternatively linked to polymers with carboxylated dimethyl maleic acid (CDM) bonds (Fig. 6). These bonds will be cleaved within the acidic environment in endosomes and lysosomes, which will expose the positively charged amino groups of the polymer backbone and trigger the influx of H^+^ and Cl^−^, resulting in the elevation of endosomal osmotic pressure and the inflow of H_2_O. The import of H^+^, Cl^−^ and H_2_O causes the destabilization and swelling of the endolysosomal membrane, which are benefits of the ‘proton sponge effect’^193^ and ‘increased colloidal osmotic pressure’.^120^ In addition, siRNAs are covalently conjugated to the polymer backbone by disulfide linkage, which will also be degraded in the environment with glutathione, releasing siRNA into the cytoplasm. Increased endolysosomal escape was accomplished by selective activation^112^ in endosomes and lysosomes, which ensures an effective interaction with other membranes before endocytosis is prevented.

For the second-generation DPC (DPC 2.0) platform, siRNA is modified with cholesterol instead of being conjugated with the polypeptide (melittin-like peptide, a kind of membrane-lytic peptide, MLP) (Figs. 5b and 6). It is worth mentioning that efficacious siRNA delivery could be achieved following the intravenous coinjection of GalNAc-modified MLP and cholesterol-conjugated siRNA (chol-siRNA). In addition, chol-siRNA can also be used in combination with GalNAc and PEG-masked PBAVE,^189^ a formulation that constitutes generation 1.5 of DPC technology. Regrettably, although biomarkers were reduced by more than 99% in HBV patients receiving a single dose of ARC-520, an siRNA therapeutic employing DPC2.0 technology, Arrowhead stopped the clinical development of this drug because one monkey died during the long-term toxicity evaluation. In addition, clinical studies of two other DPC2.0-based siRNA therapies were discontinued, and Arrowhead transferred their delivery platform from DPC to TRiM™ (Targeted RNAi Molecule) (Fig. 5c). The TRiM™ platform comprises a highly potent RNA trigger (siRNA), targeting ligands, linkers, and structures that enhance pharmacokinetic performance if needed. The targeting moiety is covalently conjugated to the siRNA directly. The targeting ligands can be selected from GalNAc, RGD motifs (ligands of integrin αvβ3 and αvβ5) and the αvβ6 ligand, which are designed to transport siRNA to hepatocytes, cancer cells and lung epithelial cells, respectively. These molecules can trigger efficacious gene silencing in the targeted tissue and potentially may reduce the risk of intracellular metabolite accumulation, thus reducing the in vivo toxicity.

#### DPC or TRiM employed therapeutics

ARC-520^194,195^ is a DPC2.0-based siRNA therapeutic containing two siRNAs. siHBV-74 and siHBV-77 elicited the greatest level of gene knockdown among ~140 candidates. Coadministration of these two siRNAs showed further gene knockdown and could be used to target 99.64% of all genotypes of HBV transcripts. Thus, ARC-520 containing these two siRNAs was proposed to combat HBV infection. In preclinical studies,^190^ ARC-520 significantly inhibited the expression of the 2.4/2.1 kb preS1/S transcripts and the 3.5 kb transcripts and reduced HBsAg levels in a dose-dependent manner, which was well maintained for ~1 month in HBV-transgenic mice.

A phase 1 study (NCT01872065) of ARC-520 showed that it was well tolerated at a dose of up to 2 mg/kg after a single dosing of ARC-520 intravenously. There was no difference between the placebo and ARC-520 groups with respect to adverse event frequency and severity. Only mild or moderate adverse events were observed. In another phase 1 study (NCT02535416), healthy adult volunteers were administered ARC-520 from 4 to 6 mg/kg at varying infusion rates, and no treatment-emergent adverse events were observed. A phase II study of ARC-520^194^ (Heparc-2001) demonstrated that HBsAg was markedly decreased in treatment-naïve and HBeAg-positive patients (Fig. 5k); however, if the patients were HBeAg-negative or previously had received treatment with nucleos(t)ide viral replication inhibitors (NUCs), the therapeutic effect was significantly reduced. More importantly, this study observed that HBsAg could be expressed from integrated HBV DNA in the host genome, in addition to expression from cccDNA. These findings uncovered an underrecognized source of HBsAg, leading to changes in the trial design and expectations for the end point of new treatments for chronic HBV.

ARC-521, another siRNA therapeutic based on the DPC2.0 delivery system, was designed as a complement to ARC-520, targeting HBV mRNA transcripts from both cccDNA and integrated DNA. This drug is expected to be most suitable for patients who have lower levels of viral cccDNA. In a clinical trial (NCT02797522) of ARC-521, healthy volunteers received single escalating doses of 0.6, 1.0, 2.0, 4.0, 5.0 and 6.0 mg/kg ARC-521,^196^ and chronic HBV patients received up to three doses (Q28 days) of ARC-521 at multiple dose levels of 2.0, 4.0 and 6.0 mg/kg. All participants were pretreated with oral antihistamine and acetaminophen 2 h prior to ARC-521 dosing. No deaths or dropouts due to AEs or SAEs were reported in healthy volunteers. No infusion reactions or laboratory abnormalities were reported as adverse effects, and no clinically significant alanine transaminase (ALT) elevations were reported in healthy volunteers. One SAE of elevated transaminases occurred in a patient at one month after a single ARC-521 dose, which may have been due to viral flare secondary to fluctuating HBV DNA and NUC nonadherence. In addition, the expression of HBsAg and HBV DNA was efficiently reduced in response to ARC-521. This effect can be elevated by combination with NUCs.

ARC-AAT was developed to treat alpha-1 antitrypsin deficiency that severely damages the liver and lungs. Two clinical trials (NCT02363946 and NCT02900183) were performed to evaluate the safety and treatment effect on intrahepatic and circulating AAT levels after ARC-AAT injection. Unfortunately, deaths were reported at the highest dose in a nonhuman primate toxicology study of ARC-520. As a result, Arrowhead stopped all three programs based on the DPC2.0 or EX-1 delivery system. Instead, Arrowhead launched programs based on the TRiM™ platform, including ARO-AAT and ARO-HBV (Table 1, Fig. 7). According to the data disclosed by Arrowhead, with the exception of being well tolerated at all doses tested, three monthly doses of 300 mg ARO-AAT^197^ led to the suppression of AAT in serum to a level below quantitation in all subjects. In addition, this suppression was constant for more than 14 weeks, suggesting that it is feasible for patients to receive dosing quarterly or even less frequently. ARO-HBV employs two siRNAs that reduce all measurable viral products and interfere with the upstream reverse transcription process in HBV patients. Data from a phase 1/2 study (NCT03365947)^198^ showed that a single dose or multiple doses of up to 400 mg of ARO-HBV was well tolerated in the patients and volunteers. Furthermore, patients receiving 3-month doses of ARO-HBV and entecavir or tenofovir showed up to −3.8 log10 HBsAg reduction. More comprehensive studies are ongoing.

### GalNAc–siRNA conjugates

#### Characteristics of GalNAc–siRNA conjugate platforms

Although patisiran, a liposome-based RNAi formulation, reached commercial application, Alnylam still stopped all other liposome-based programs, as the GalNAc–siRNA conjugate delivery platform is superior to that of liposomes regarding both potency and safety for the liver-targeted delivery of siRNA. GalNAc is a ligand of the asialoglycoprotein receptor (ASGPR), which is an endocytic receptor that is highly and specifically expressed on the membrane surface of hepatocytes (~500,000 copies/cell) but barely expressed by other cells.^199^ ASGPR- and clathrin-mediated endocytosis can efficiently transport galactose-derived ligands from the cell surface to the cytoplasm. In this process, ASGPR is presented on the cell surface within 15 min. Early structure–activity studies revealed that tetravalent and trivalent ligands exhibit orders of magnitude higher affinity than monovalent and bivalent ASGPR when the spacing is ~20 Å. Hence, trivalent or tetravalent GalNAc moieties are covalently conjugated to siRNA with a proprietary linker structure (Figs. 5d and 6). Targeting ligands are placed at the 3′ and 5′ ends of the siRNA sense strand for Alnylam and Arrowhead formulations, respectively.^31,200^ In contrast, based on the specific DsiRNA structure, four GalNAc residues are positioned at the four unpaired tetraloop-hairpin nucleotides of Dicer on the sense strand (Figs. 3 and 5g).^201^

Evidence^200^ showed that subcutaneously administered GalNAc–siRNAs achieved better performances than intravenously administered GalNAc–siRNAs. It is hypothesized that subcutaneously administered siRNAs need to cross connective tissue and capillary and lymphatic endothelial cells to accumulate in circulation. This process changes the pharmacokinetic profile of GalNAc–siRNA. Higher siRNA explosion in the liver was achieved with subcutaneously injected than intravenously injected GalNAc–siRNA because intravenously injected siRNA was excreted quickly from circulation to the kidneys.^49^ Meanwhile, the cellular entry of GalNAc–siRNA is mediated by ASGPR via a naturally existing mechanism, which constitutes a safer way for siRNA delivery than liposomes, which may trigger detrimental lipid interactions between the cell membrane and the liposome. As a result, GalNAc–siRNA conjugates show excellent pharmaceutical properties, such as allowing subcutaneous administration, with high tissue-targeting specificity, a higher therapeutic index and minimal or limited AEs. More importantly, these conjugates show an extremely long duration of action when combined with enhanced stabilization modification chemistry, e.g., ESC or ESC-plus (Fig. 3), supporting quarterly and even twice-yearly dosing of these RNAi therapeutics.

In addition to Alnylam, other biotechnological companies have also established GalNAc–oligonucleotide conjugate platforms, e.g., Arrowhead,^202^ Dicerna,^203^ Arbutus,^188,204^ Silence,^205^ Ionis,^206^ Wave Life Sciences,^207^ Regulus^208^ and Suzhou Ribo Life Science^209^ (Fig. 6). Moreover, Mascitti and colleagues^210^ from Pfizer developed a variety of GalNAc analogs as alternative ligands for ASGPR. Compared to the GalNAc-based conjugate, conjugating a compact and stable bicyclic bridged ketal to small-molecule cargo with an oxanorbornadiene linkage could achieve increased ASGPR-mediated cellular uptake and prolonged retention. A proprietary stereopure anti-ApoC3 ASO developed by Wave Life Sciences also achieved efficient liver-targeted delivery of ASO and showed robust gene repression.^211^

GalXC™^94^ (Fig. 5g) is a proprietary RNAi delivery platform engineered by Dicerna Pharmaceuticals that facilitates the establishment of plentiful therapeutic pipelines. Dicer-substrate siRNA (DsiRNA) is composed of a sense strand with tetraloop hairpin, a constant sequence, and a 21–23 nt antisense strand complementing the target mRNA. Multivalent GalNAc moieties are linked to DsiRNA at the region of the tetraloop hairpin. The prominent feature of this platform is the introduction of a constant complementary structure in the siRNA sense strand that makes it difficult to be loaded by RISC. Thus, sense strand-mediated off-target effects might be efficiently erased. In addition, a region of DsiRNA was engineered into the GalXC™ molecules to provide additional pharmaceutical functionality. For example, this modification could enhance silencing activity, inducing higher affinity for serum albumin than no modification and improving the circulating half-life of GalXC™ molecules. There are no fixed modification strategies for GalXC™. Several representative modification patterns are shown in Fig. 3, in which a few DNA nucleotides are introduced into the DsiRNA. Another typical characteristic is to use naturally occurring or FDA-approved modification motifs to reduce toxic side effects. As a result, high efficacy (ED_50_ < 0.3 mg/kg) and good tolerability (no clinical adverse events recorded when administered at 100 mg/kg weekly for 6 weeks) can be achieved.

#### GalNAc–siRNA conjugate-based therapeutics

Many GalNAc-conjugated oligonucleotide therapeutics are undergoing clinical studies (Table 1, Fig. 7). Recently, only four months after receiving new drug application (NDA) from Alnylam, the FDA-approved GIVLAARI™ for the treatment of AHP,^99^ a genetic disorder resulting in the buildup of toxic porphyrin molecules that are formed during the production of heme (which helps bind oxygen in the blood). GIVLAARI™, an aminolevulinic acid synthase 1 (ALAS1)-targeting RNAi therapeutic, is the first GalNAc-conjugate RNAi therapeutic worldwide. As mentioned above, ESC modification confers siRNA more stability, which allows quarterly dosing at significantly lower dosages. According to the phase 1 clinical data disclosed by Alnylam,^9^ givosiran dosed at 2.5 mg/kg once-monthly significantly reduced the levels of ALAS1 mRNA, delta aminolevulinic acid (ALA) and porphobilinogen (PBG), with higher than 80% inhibition efficiency, which was accomplished with a 79% decrease in the mean annualized attack rate compared to that of the placebo. In the ENVISION phase 3 study, patients receiving givosiran showed a 74% mean reduction in the annualized rate of composite porphyria attacks compared to those receiving placebo, with an acceptable overall safety and tolerability profile (Fig. 5i).^10^

In addition, inclisiran and lumasiran have been submitted to the FDA for new drug commercialization evaluation, and fitusiran (ALN-AT3) and vutrisiran (ALN-TTRsc02) are undergoing phase 3 clinical trials (Table 1, Fig. 7). Fitusiran was developed for the treatment of hemophilia and rare bleeding disorders (RBDs) and functions by inhibiting the expression of antithrombin (AT). This drug employs the ESC-GalNAc delivery platform developed by Alnylam. A phase 2 study (NCT02554773)^94,212,213^ showed that 50 or 80 mg of fitusiran (lower than 2.5 mg/kg) per month resulted in an antithrombin reduction of 78–88% from baseline. The phase 2 trial of inclisiran (NCT02963311),^38,214–216^ an siRNA targeting proprotein convertase subtilisin kexin type 9 (PCSK9), also exhibited potent and durable gene silencing and a reduction in low-density lipoprotein cholesterol (LDL-C) (Fig. 5j). Lumasiran is an RNAi modality for treating primary hyperoxaluria type 1 (PH1) that functions by inhibiting the expression of glycolate oxidase. Phase 1/2 studies of lumasiran revealed a mean maximal reduction in urinary oxalate (Uox) of 75% relative to baseline across all cohorts (1 mg/kg monthly, 3 mg/kg monthly, and 3 mg/kg quarterly; *N* = 20). All lumasiran-treated patients experienced a mean maximal decrease of 77% in the ratio of urinary oxalate to creatinine, an additional measure of oxalate reduction that addresses the variability inherent in 24-h urine collections. An acceptable safety and tolerability profile was also observed in phase 1/2 studies. Moreover, a phase 1 clinical investigation of vutrisiran (NCT02797847)^217^ showed that the reductions in serum TTR were well maintained for as long as 1 year after a single dose of vutrisiran for all six dose groups from 5 to 300 mg.

Cemdisiran, ALN-AAT02, ARO-HBV and DCR-PHXC are undergoing phase 2 trials (Table 1, Fig. 7). Cemdisiran (Fig. 4f) and ALN-AAT02 are two programs developed by Alnylam for the treatment of complement-mediated diseases and AAT deficiency-associated liver disease (alpha-1 liver disease) and function by targeting the C5 component of the complement pathway and alpha-1 antitrypsin (AAT), respectively. Cemdisiran and ALN-AAT02 utilize the ESC-GalNAc delivery platform and the ESC-Plus (ESC+)-GalNAc delivery platform, respectively, developed by Alnylam (Figs. 3, 5d and 6). ARO-HBV and nedosiran (DCR-PHXC) were developed by Arrowhead and Dicerna for curing hepatitis B and primary hyperoxaluria (PH), respectively. DCR-PHXC can inhibit the expression of lactate dehydrogenase A (LDHA), thus blocking the excess production of oxalate, leading to a cure for all three genetic forms of PH (type 1, 2 and 3), a rare, inherited, liver metabolic disorder. The current treatment approaches for PH are either poorly effective or highly invasive.^218^ In an undergoing study of DCR-PHXC-101 in healthy volunteers and patients^219^ (phase 1, NCT03392896), the patients were divided into three cohorts that received different doses of DCR-PHXC via subcutaneous administration. The 24-h Uox values for three of the five patients who received 1.5 mg/kg of DCR-PHXC reached near-normalization levels at one or more postdose time points, and their mean maximal 24-h Uox reduction was 51% (range: 28–72%). When the dose was doubled, the 24-h Uox values for 4 out of 5 subjects reached normalization at one or more postdose time points, and their mean maximal 24-h Uox reduction was 71% (range: 62–80%). For the third cohort receiving 6 mg/kg DCR-PHXC, the mean maximal 24-h Uox reduction reached 76% (range: 58–100%). The 24-h Uox values were not disclosed because the values for 2 patients did not return to within 80% of the lowest baseline 24-h Uox measurement (Fig. 5n).

AMG 890 (formerly ARO-LPA), ARO-ANG3, ARO-APOC3, ARO-AAT (Fig. 5o), DCR-HBVS, ALN-AGT01 and AB-729 were evaluated in phase 1 clinical trials (Table 1, Fig. 7). These therapeutics were developed to treat cardiovascular disease, dyslipidemia, hypertriglyceridemia, alpha-1 liver disease, hepatitis B, hypertension and hepatitis B and function by targeting apolipoprotein A (Apo(a)), angiopoietin-like 3 (ANGPTL3), apolipoprotein C3 (ApoC3), AAT, the HBV gene, angiotensinogen (AGT) and the HBV gene, respectively. Moreover, many other candidates have been explored preclinically for liver-related diseases, e.g., ALN-HBV02 (VIR-2218), SLN360, SLN226, RBD1016, RB-HLP002 and DCR-A1AT.^2^

### LODER™ and siG12D-LODER

LOcal Drug EluteR (LODER™)^220,221^ is a biodegradable polymeric matrix derived from poly(lactic-co-glycolic) acid (PLGA) with a small cylindrical rod ~1 nm in diameter and ~5 nm in length (Figs. 5e and 6). According to Silenseed, LODER™ is the first siRNA delivery system that allows RNAi drugs to be directly delivered into solid tumors. LODER™ enables the slow and stable release of locally delivered drugs. The siRNA in LODER™ was protected from degradation when incubated in phosphate buffer solution or in mouse liver tissue homogenate for over 2 months.

siG12D-LODER is a G12D-mutated KRAS-targeting siRNA therapeutic entrapped in LODER™. Preclinical studies performed in rats and mice showed robust tumor treatment effects at a dose of 0.32 mg/kg. According to preclinical evaluation data collected from 192 rats, siG12D-LODER revealed ideal safety profiles in all animals receiving repeated subcutaneous doses of the studied formulation at days 1, 14 and 28.^222^ In phase 1/2a studies (NCT01188785 and NCT01676259),^223^ siG12D-LODER in combination with chemotherapy (gemcitabine or FOLFIRINOX) was used as a first-line treatment for inoperable locally advanced pancreatic cancer (LAPC) patients. Three escalating dose cohorts (0.025, 0.75 and 3.0 mg) were employed in this study. The main adverse events were grade 1 or 2 in severity (89%), and serious adverse events (SAEs) occurred in five patients. Tumor progression was not observed for 12 patients according to the CT scan data (Fig. 5l). Stable disease was achieved in 10 of 12 patients, and partial response was observed in 2 patients. The median overall survival of siG12D-LODER-treated patients was 15.12 months, and the 18-month survival reached 38.5% of patients.

Based on LODER™ delivery technology, Silenseed developed si-PT-LODER^224^ aiming to cure prostate cancer and si-GBMT-LODER as a treatment for brain cancer, which are in the preclinical and research stages, respectively.

### Other platforms

Polymers^17,114,115,225–233^ (e.g., PEI,^231^ PTMS,^115^ GDDC4^114^ and PAsp(DET)^232^) constitute additional important siRNA delivery platforms. Similar to LNPs, polymers can achieve high cellular uptake and endosomal escape because they are positively charged. Besides LODER™, the cyclodextrin-based RONDEL™ is another polymer platform that was developed by Calando Pharmaceuticals and was once investigated in a phase I clinical study^234,235^ (Fig. 6). Repeatability, manufacture-controllability, safety and biodegradability are the key issues that researchers need to pay attention to when studying polymers. Therefore, designing simple, biodegradable, targeted or naturally occurring polymers is a potential development direction for polymer-based platforms.

In addition, some other systems are also important for siRNA therapeutic development, which include (i) the EDV™ nanocell platform developed by EnGeneIC;^236^ (ii) the HKP delivery system employed by Sirnaomics, Inc.^237^ (Fig. 6); (iii) extracellular vesicles or exosomes derived from various cell types (Fig. 5f, m);^16,238,239^ (iv) peptides;^240–243^ (v) dendrimers;^244–246^ (vi) RNA nanoparticles (e.g., three way-junction, 3WJ);^247–254^ and (vii) inorganic nanoparticles^229,255^ (Fig. 6). These platforms also exhibited satisfactory delivery efficiency in the preclinical and/or clinical stages.

To date, hepatocyte-targeted delivery of siRNA has been well resolved by leveraging GalNAc–siRNA conjugates. In the following days, researchers will develop extrahepatic siRNA delivery strategies. Recently, central nervous system (CNS) and eye-targeted delivery platforms have been established by conjugating a lipophilic moiety to the siRNA, according to the descriptions in Alnylam’s patent (WO2019217459A1).^2,256^ Arrowhead successfully delivered siRNA to renal cancer cells and lung epithelial cells probably by employing RGD motifs or small molecules as the targeting moieties.^2^ In addition, various lipids or fatty acids have also been used to modify oligonucleotides, including siRNA^257–260^ and ASOs,^261–263^ to achieve robust gene silencing in extrahepatic tissues, e.g., heart, lung, kidney, skeletal muscle, brain, etc. This progress remarkably extends the diseases that can be treated by RNAi therapeutics.

## Conclusions

Due to the mechanism of action of siRNA, this molecule can be used to target nearly all disease-related genes of interest. The duration of drug research and development is also much shorter than that for small molecules, monoclonal antibodies and proteins. After the successful commercialization of ONPATTRO^®^ and GIVLAARI™ for the treatment of rare diseases, siRNA therapy will achieve another milestone soon for treating common diseases, e.g., dyslipidemia, as excellent phase 3 data have been collected from several clinical studies of inclisiran.

Benefiting from long-term and constant innovations in exploring modification geometries and delivery systems, the effective dose and half-life of the siRNA modalities were reduced from milligrams to 10^−3^ mg and prolonged from minutes to months, respectively. GalNAc–siRNA conjugates with sophisticated chemical modifications enable quarterly or twice-yearly subcutaneous administration of siRNA therapeutics, which is a great achievement that had never been realized in the history of pharmaceuticals. These improvements will exert widespread and far-reaching impacts on the whole medical industry, e.g., on drug development, government management, patient treatment, payment patterns of medical insurance, financial investments from the capital market, etc.

After studying GalNAc–siRNA conjugation for hepatic delivery of siRNA, researchers will devote themselves to developing extrahepatic delivery platforms. Several important advances have been achieved for renal, CNS and ocular targeted delivery of siRNA. Therefore, state-of-the-art siRNA technologies will definitely change our life in the future.
